# An oxygen-sensitive toxin–antitoxin system

**DOI:** 10.1038/ncomms13634

**Published:** 2016-12-08

**Authors:** Oriol Marimon, João M. C. Teixeira, Tiago N. Cordeiro, Valerie W. C. Soo, Thammajun L. Wood, Maxim Mayzel, Irene Amata, Jesús García, Ainara Morera, Marina Gay, Marta Vilaseca, Vladislav Yu Orekhov, Thomas K. Wood, Miquel Pons

**Affiliations:** 1 Biomolecular NMR Laboratory, Organic Chemistry Section, Inorganic and Organic Chemistry Department, University of Barcelona, Baldiri Reixac 10-12, Barcelona 08028, Spain; 2 Department of Chemical Engineering and Department of Biochemistry and Molecular Biology, Pennsylvania State University, University Park, Pennsylvania 16802, USA; 3 Swedish NMR Centre, Gothenburg University, PO Box 465, Gothenburg SE-40530, Sweden; 4 Institute for Research in Biomedicine (IRB-Barcelona), The Barcelona Institute of Science and Technology, Baldiri Reixac 10-12, Barcelona 08028, Spain

## Abstract

The Hha and TomB proteins from *Escherichia coli* form an oxygen-dependent toxin–antitoxin (TA) system. Here we show that YmoB, the *Yersinia* orthologue of TomB, and its single cysteine variant [C117S]YmoB can replace TomB as antitoxins in *E. coli.* In contrast to other TA systems, [C117S]YmoB transiently interacts with Hha (rather than forming a stable complex) and enhances the spontaneous oxidation of the Hha conserved cysteine residue to a -SO_*x*_H-containing species (sulfenic, sulfinic or sulfonic acid), which destabilizes the toxin. The nuclear magnetic resonance structure of [C117S]YmoB and the homology model of TomB show that the two proteins form a four-helix bundle with a conserved buried cysteine connected to the exterior by a channel with a diameter comparable to that of an oxygen molecule. The Hha interaction site is located on the opposite side of the helix bundle.

## Abstract (web summary)

Classical toxin–antitoxin systems in bacteria are based on silencing of a toxin by an antitoxin that, when inactivated, releases the toxin, resulting in a change in metabolism. Here, the authors characterize an oxygen-sensitive toxin–antitoxin system and discuss the implications for the role of the Hha antitoxin.

Antimicrobial resistance is a major threat to global health^1^. Biofilms are resistant communities of microorganisms attached to surfaces and encapsulated in a matrix^2^,^3^. Biofilms are involved in 80% of human bacterial infections^4^ and confer antibiotic resistance^5^.

Oxygen consumption in the biofilm generates oxygen gradients^6^,^7^ and may be linked to biofilm dispersal^8^. Selective cell death, for example, in anoxic regions, creates channels that facilitate nutrient-waste product exchange^9^,^10^,^11^ and biofilm dispersion^12^. Dispersed cells are more susceptible to antibiotics than those in biofilms, although they are also distinct from planktonic cells in terms of gene expression and pathogenicity^13^.

Classical toxin–antitoxin (TA) systems are based on silencing of a stable toxin by a labile antitoxin that, when inactivated, releases the toxin, resulting in a reduction in metabolism. TA systems modulate the generation of persister cells^14^, phage inhibition^15^ and biofilm regulation^16^.

The Hha/TomB TA system is part of the first group of TA systems identified in biofilms^17^. The overexpression of Hha (haemolysin expression modulating protein) causes cell lysis and reduction of biofilm formation as well as increases biofilm dispersal^18^. An engineered variant of Hha caused nearly complete biofilm dispersal via cell lysis^19^,^20^, and deletion of *hha* led to no dispersal of *Escherichia coli* BW25113 (ref. 20) corroborating the role of Hha in biofilm dispersal. TomB (toxin overexpression modulator in biofilms, previously known as YbaJ) inactivates Hha toxicity^18^ although the mechanism has not been elucidated.

Here we report that the TomB antitoxin activity is oxygen-dependent and that its *Yersinia* orthologue, YmoB can replace TomB as the Hha antitoxin. A single cysteine variant [C117S]YmoB is similarly active *in vivo* and promotes the oxidation of the single conserved cysteine of Hha to -SO_*x*_H (sulfenic, sulfinic and sulfonic acids) by air in the absence of any external source of reactive oxygen species. Oxidation of Hha or the introduction of a negative charge at the position of the cysteine residue, mimicking the presence of -SO_*x*_^−^, causes its destabilization and results in reduced toxicity.

The three-dimensional (3D) structure and also the Hha transient interaction site of [C117S]YmoB were determined by nuclear magnetic resonance (NMR). Relevant structural features include a buried cysteine residue located close to the Hha-binding site and connected to the opposite side of the four-helix bundle by a narrow channel with a width comparable to that of an oxygen molecule. The built-in oxygen sensor in this TA system may provide a mechanism for environmental regulation of cell activity.

Taken together, these observations suggest that Hha/TomB represent a TA system based on the inactivation of the toxin (Hha) by oxidation with molecular oxygen mediated by the antitoxin (TomB).

## Results

### TomB antitoxin activity is oxygen-dependent

**Figure 1:**
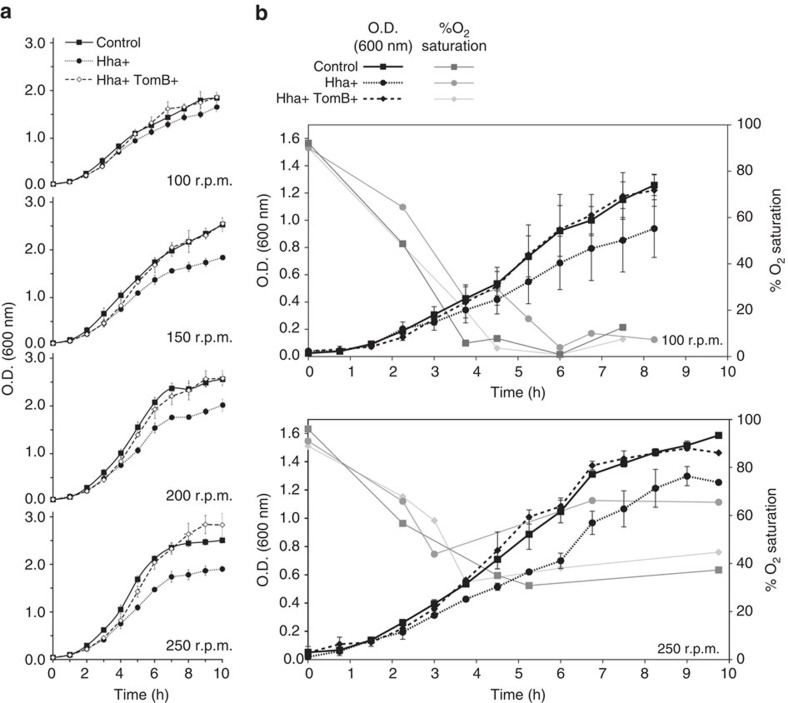
TomB antitoxin activity depends on the agitation rate. (**a**) Growth curves of *E. coli* K-12 MG1655 *Δhha* cells harbouring pCA24N-*hha* and pBAD30-*tomB* at (from top to bottom) 100, 150, 200 and 250 r.p.m. agitation rates. Control cultures with no expression of Hha or TomB (squares) were compared with cultures expressing only Hha (circles) and co-expressing both Hha and TomB (diamonds). Growth curves were measured in triplicate at 37 °C in tryptone minimal medium (TMM; 10 g l^−1^ tryptone and 2.5 g l^−1^ NaCl). Error bars represent the sample s.d. (**b**) Growth curves and simultaneous measurements of the oxygen saturation during culture growth at 150 r.p.m. (top) and 250 r.p.m. (bottom) agitation rates.

We tested the hypothesis that the Hha-TomB pair was an oxygen-dependent TA system by measuring *E. coli* growth at increasing agitation rates (Fig. 1). Overexpression of Hha has a toxic effect in minimal medium, resulting in a decrease of the growth rate and the cell density in the stationary phase. Growth in rich medium showed an even higher toxicity^18^. Simultaneous overexpression of Hha and TomB in minimal medium results in biphasic growth gradually evolving from the slow growth rate observed when Hha is overexpressed in the absence of TomB to the faster growth observed in the controls not expressing Hha. A significant antitoxin effect of TomB was observed after ∼4 h, and after 6 h, cultures overexpressing both Hha and TomB reached the same cell densities as the controls.

Experiments at the two extreme agitation rates were repeated while simultaneously measuring oxygen saturation, using an oxygen-sensitive polarographic electrode (Fig. 1b). Oxygen levels reflect the balance between air uptake, enhanced by fast agitation, and oxygen consumption by the metabolic activity of the bacteria. At an agitation rate of 100 r.p.m., the oxygen saturation levels of the control and Hha+TomB expressing cultures reached a minimum of 1% while at an agitation rate of 250 r.p.m., the oxygen levels were always higher than 30%. Oxygen levels increased when the cultures approached the stationary phase. The longer lag time for the antitoxin activity at slow agitation rates is consistent with oxygen being required for TomB activity as an antitoxin.

At the agitation rate of 250 r.p.m., the oxygen levels of the cultures overexpressing only Hha were always higher than the controls, reflecting a lower oxygen consumption in the presence of the toxin. At 100 r.p.m., cultures expressing Hha+TomB or only Hha reached similar oxygen levels (10% saturation) in late stationary phase in spite of the fact that the cell density, as measured by the turbidity at 600 nm, was lower in the cultures expressing only the toxin. However, in these cultures, the number of colony forming units (CFU) was lower than in those expressing also the antitoxin (Supplementary Table 1). These observations suggest that 10% oxygen saturation is enough to enable TomB antitoxin activity. Interestingly, the observation that the number of CFU is higher in the samples overexpressing the two proteins (Hha+TomB) than in those only expressing Hha rules out the possibility that the observed toxicity of Hha is only related to the metabolic stress associated with protein overexpression and confirms that the toxicity of Hha can be compensated by TomB.

These observations suggest an oxygen-dependent TA mechanism that we investigated in detail by combining chemical biology and structural studies.

### YmoB protects *E. coli* cells from Hha toxicity

**Figure 2:**
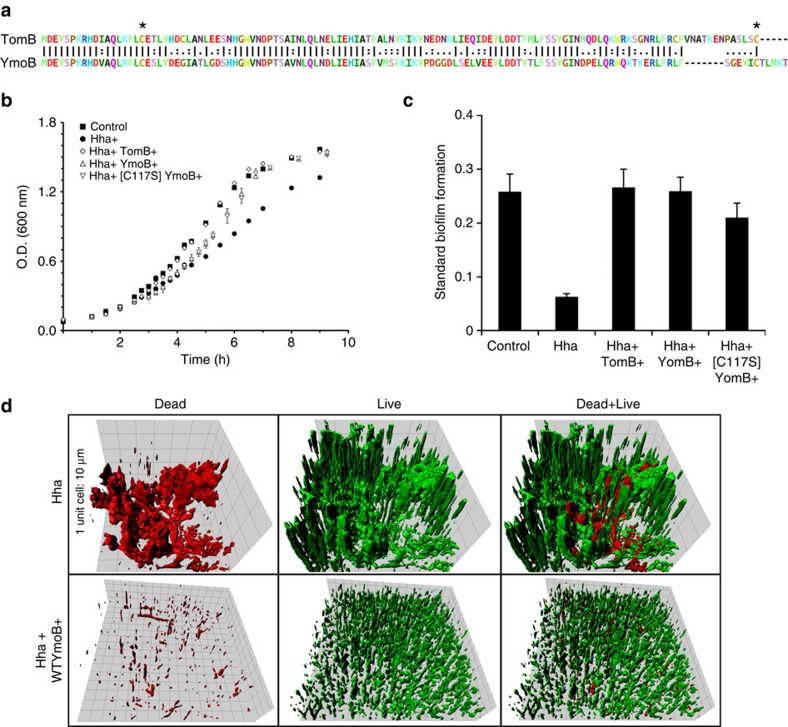
*Yersinia'*s YmoB protects *E. coli* from toxic effect of Hha. All experiments were carried out using *E. coli* K-12 MG1655 *Δhha* cells harbouring pCA24N-hha and pBAD30-*tomB*, pBAD30-*ymoB* or pBAD30-*ymoB(C117S)*, at 37 °C and in TMM. (**a**) Sequence alignment of TomB/YmoB proteins. Asterisks highlight the position of conserved cysteine residues at positions 18 and 117. (**b**) Growth curves of control cultures with no expression of a TA pair of proteins (squares) were compared with cultures overexpressing: Hha (circles), Hha and TomB (diamonds), Hha and YmoB (triangles up) or Hha and [C117S]YmoB (triangles down). Experiments were performed in duplicate, and the error bars are the sample standard deviation. (**c**) Microtitre plate biofilm assay. Total biofilm formation was measured at OD_540nm_ and was standardized as OD_540nm_/OD_620nm_. Experiments were performed in duplicate, and the error bars are the sample standard deviation. (**d**) Representative IMARIS (BITplane, Zurich, Switzerland) images of flow cell biofilm with Hha overexpression (up) and Hha and YmoB co-expression (down). Dead cells (left) are shown in red and live cells (centre) in green, while superimpositions of dead and alive cells are shown on the right. OD, optical density.

A non-redundant collection of 250 TomB homologues was obtained by BLAST search using as a query the TomB sequence from *E. coli* K-12 strain (D64776). The sequences (Supplementary Fig. 1), representative of the YbaJ-superfamily, contain from one to five cysteines. Cysteine 18 (in 244 sequences) and a second cysteine located towards the C-terminus (235/250 sequences) are most conserved. Attempts to obtain suitable constructs to carry out structural studies of TomB failed, so we focused on its *Yersinia's* homologue YmoB, which has 58.1% pairwise identity, 73.6% pairwise similarity and contains the two most conserved cysteines (C18 and C117, Fig. 2a). Its single cysteine variant [C117S]YmoB proved suitable for structural determination by NMR, as shown below.

We first explored if YmoB and [C117S]YmoB could functionally replace TomB in preventing Hha-associated toxicity in *E. coli* in planktonic cells and in biofilms. Planktonic cell cultures overexpressing Hha and either of the two YmoB variants reached the same cell density of the controls not expressing Hha or overexpressing both Hha and TomB (Fig. 2b), although the antitoxin activity of the YmoB variants showed a longer lag time than TomB. The C117S substitution had no significant effect on YmoB antitoxin activity in planktonic cells.

Overexpression of Hha causes a fourfold decrease of standard biofilm formation (Fig. 2c). The co-expression of TomB or YmoB totally restored biofilm formation, although the C117S variant was slightly less efficient. Regarding biofilm morphology, Hha overexpression leads to amorphous biofilms with a higher percentage of dead cells (Live/Dead cells ratio of 5.6±1.4, quantitative analysis by COMSTAT^21^ software). Instead, when YmoB was co-expressed, the Live/Dead cell ratio significantly increased to 35.4±8.5 and the biofilm was composed by microcolonies uniformly spread on the entire surface (Fig. 2d). Thus, YmoB or [C117S]YmoB can functionally replace TomB as the Hha antitoxin in *E. coli*.

### [C117S]YmoB enhances Hha oxidation *in vitro*

Hha has a single cysteine residue (C18) highly conserved in the Hha-family of proteins. We hypothesized that the oxygen-dependent antitoxin activity of TomB and [C117S]YmoB towards Hha could be related to the oxidation of this conserved cysteine residue.

In addition to their capacity to form disulfide-bonds, cysteine thiol groups can be oxidized to sulfenic (-SOH), sulfinic (-SO_2_H) and sulfonic (-SO_3_H) acids^22^. Sulfenic acid formation is reversible but sulfinic and sulfonic acid are irreversibly formed and result in the introduction of a negative charge. Hha has an unusual high proportion (33%) of charged residues and its structure is highly sensitive to electrostatic effects^23^. The additional charge introduced by cysteine oxidation could destabilize Hha and prevent its inherent toxicity.

**Figure 3:**
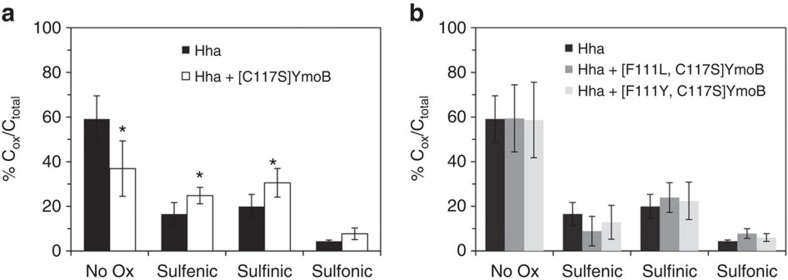
[C117S]YmoB enhances the oxidation of Hha. The bars represent the percentage of peptide spectrum matches (PSM) to the various oxidation states relative to the total PSM of cysteine-containing peptides in the mass spectra of trypsin-treated samples of Hha. Parallel experiments were performed by incubating Hha for 30 min without any added YmoB variant, or in the presence of (**a**) [C117S]YmoB, or (**b**) [F111L,C117S]YmoB or [F111Y,C117S]YmoB. The experiments were run in triplicate and each sample was analysed in duplicate. The error bars are the standard deviations. Asterisks mark statistically significant deviations according to a Student *t*-test with *P*=0.02. [C117S]YmoB enhanced oxidation of YmoA was measured under different conditions and is shown in Supplementary Fig. 2.

To investigate the effect of YmoB on the oxidation of C18 of Hha *in vitro,* we used mass spectrometry. Identical samples of Hha were incubated in the presence and in the absence of [C117S]YmoB for 30 min. Hha was isolated by size exclusion chromatography (SEC), digested with trypsin and analysed by mass spectrometry to identify cysteine-containing peptides in their reduced and oxidized (-SO_*x*_H) forms. Figure 3a shows a decrease in the reduced form and an increase in the oxidized forms of Hha in the presence of [C117S]YmoB. The differences with the untreated Hha samples are statistically significant according to a Student *t*-test with *P*=0.03 (for sulfonic acid) and *P*=0.02 (reduced and other oxidized forms). Variants of YmoB in which F111 had been mutated showed no effect (Fig. 3b). The choice of the phenylalanine mutants was based on the structural studies described below. Interestingly, even in the absence of YmoB, Hha contains a significant proportion of peptides with oxidized forms of cysteine suggesting that the effect of YmoB is to enhance its spontaneous oxidation. The Hha homologue in *Yersinia*, the protein YmoA, showed similar oxidation effects (Supplementary Fig. 2).

### A negative charge at the cysteine position destabilizes Hha

**Figure 4:**
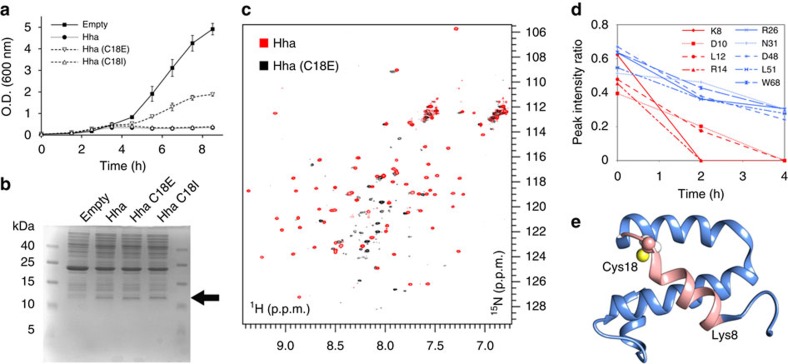
A negative charge in position 18 of Hha destabilizes the toxin. The negative charge introduced by cysteine oxidation was mimicked by the C18E mutation. (**a**) Growth curves of *E. coli* BW25113 *Δhha* cells harbouring: pCA24N (squares), pCA24N-hha (circles), pCA24N-*hha*(C18E) (triangles down) and pCA24N-*hha*(C18I) (triangles up). Each data point represents the mean of three independent cultures, and error bars denote s.e.m. (**b**) *E. coli* BW25113 *Δhha* cell lysates after 4 h growth harbouring: pCA24N (empty), pCA24N-*hha* (Hha), pCA24N-*hha*(C18E) (Hha C18E) and pCA24N-*hha*(C18I) (Hha C18I). Arrow denotes Hha and its mutated variants. For each strain, 25 μg of total protein was loaded on 18% SDS-PAGE. Experiments performed at 37 °C in LB medium. (**c**) ^1^H–^15^N HSQC NMR spectra of Hha (red) and [C18E]Hha (black). The reduced spectral dispersion in the spectra of the mutated protein indicates a loss of tertiary structure. (**d**) Changes in NMR peak intensity of native Hha treated with hydrogen peroxide. The intensity changes of peaks in helix 1 are indicated in red, while peaks in other regions are shown in blue. (**e**) Hha structure with helix 1 and cysteine 18 highlighted. SDS–PAGE, SDS–polyacrylamide gel electroporesis.

We mimicked the effect of adding a negative charge at the cysteine position by mutating it to glutamic acid, and we measured the growth of *E. coli Δhha* expressing the [C18E]Hha variant. As controls, we used wild-type Hha and a previously studied C18I variant^24^. While [C18I]Hha retains its capacity to act as a toxin, the presence of a negative charge at this position substantially reduces the toxicity of the C18E variant (Fig. 4a).

The expression levels of wild type, C18I and C18E variants of Hha are comparable after 4 h of induction at 37 °C (Fig. 4b). However, when [C18E] Hha was overexpressed and purified, this variant showed a complex NMR spectrum with low dispersion and non-uniform peak intensities typical of a denatured protein (Fig. 4c), while the C18I mutant had been previously shown to be well folded^24^. Thus, the introduction of a negative charge at position 18 destabilizes the structure of Hha. A similar effect had been previously observed in Hha, when changing the electrostatic balance by the removal of a negative charge via the substitution E53Q, which resulted in complete protein denaturation^25^.

Hha was also treated with hydrogen peroxide *in vitro* to induce its oxidation. The NMR spectra of ^15^N-labelled Hha showed a clear decrease in intensity over time after the addition of H_2_O_2_ (Fig. 4d). The most-affected region is centred in a helical segment next to the cysteine residue (Fig. 4e). Helix 1 contains a methionine residue that can also be oxidized. The loss of intensity indicates a structural change from the native form. A dynamic molten-globule type structure or soluble aggregates are two of the obvious possibilities. Dynamic light-scattering experiments showed no significant variation after treatment with H_2_O_2_ suggesting the absence of aggregation (Supplementary Fig. 3).

Taken together, the *in vivo* and *in vitro* results confirm that YmoB variants or TomB act as antitoxins by enhancing the inactivation of Hha by oxidation.

### 3D structure of [C117S]YmoB

To gain molecular level insight into the role of [C117S]YmoB in the oxidation of Hha, we decided to determine its 3D structure. No structure of any member of the YbaJ-superfamily is yet known.

Although the [C117S]YmoB variant provided NMR spectra of enough quality to complete the backbone assignment (Supplementary Fig. 4), slow time evolution hindered the assignment of the side chains associated NOE (Nuclear Overhauser Effect) cross-peaks required for structure determination. Therefore, the suite of NMR experiments needed to determine the 3D structure of [C117S]YmoB were carried out in interleaved mode using non-uniform sampling and targeted acquisition^26^. The 3D structure was determined using 1349 NOE/distance, 198 dihedral angle and 36 hydrogen bond restraints with UNIO'10/CYANA 2.1 software^27^,^28^,^29^ and final refinement in the presence of explicit water with CNS 1.2.1 software^30^,^31^. Refinement statistics are summarized in Table 1. The NMR assignment and the structural models have been deposited in the RCSB PDB with accession numbers RCSB104115 and 2MN2. Details of the process, experiments and NMR calculation are presented in the Supplementary Tables 2 and 3.

**Figure 5:**
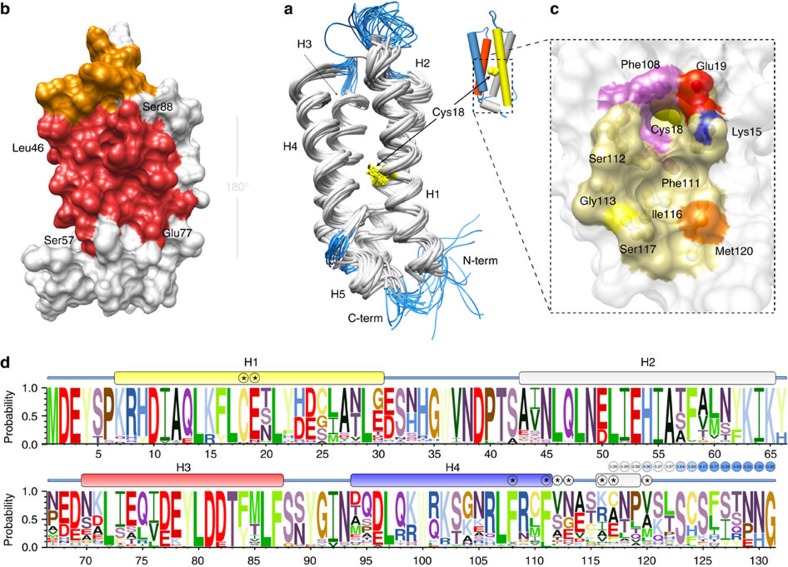
NMR structure of [C117S]YmoB as a model for TomB structure. (**a**) Superimposition of 20 lowest energy models. Helical regions (H1: 7–30; H2: 43–65; H3: 70–87; H4: 94–111; and H5: 116–119) are shown in white and non-structured regions in blue. C18 is coloured yellow. The same colour is used for H1 in the topological representations. Helices H3 (negatively charged) and H4 (positively charged) are represented in red and blue, respectively. (**b**) Surface representation of the residues broadened in the presence of paramagnetically labelled Hha. Those located in helices H2 and H3 are shown in red. Additional affected residues, (mainly in loops) are marked in orange (see Supplementary Fig. 4). The structures in **a**,**b** are rotated 180°. Most of the residues contacting Hha are located in one side of the structure. (**c**) Expanded view of the pocket and channel defined by conserved residues F108 and F111 (purple), K15 (blue), E19 (red) and S117 (C117 in wild-type YmoB, yellow) connecting the surface with the location of the sulfur atom of C18. M120, also susceptible to oxidation and close to C117, is shown in orange. The entry of the channel is located in the opposite face from the Hha-binding site. S117 is occupied by cysteine in the wild-type form of YmoB. (**d**) Logo representation of the sequence variability within the YbaJ-superfamily. Secondary structure of [C117S]YmoB is represented. An asterisk highlights residues forming the pocket surrounding C18. Some of the sequences terminate after position 116. The proportion of sequences that extended to a given position is shown and colour-coded in circles at the C-terminal end of the logo representation.

Figure 5a shows the superposition of the 20 lowest energy models, which have a root mean square deviation considering all backbone heavy atoms of 1.89±0.45 Å, and 0.87±0.14 Å if only the residues in the α-helices are compared. [C117S]YmoB forms a compact antiparallel four-helix bundle connected by flexible loops. A one-turn helix H5, containing the C117 in the wild-type form, is located at the C-terminus.

A singular feature of the structure is the location of the isolated cysteine residue (C18) in helix H1, which is completely buried in the protein hydrophobic core. Consistently, C18 is completely inaccessible to iodoacetic acid.

### Transient interactions of [C117S]YmoB with Hha and YmoA

Typical TA pairs form stable complexes masking the toxin. However, addition of Hha or YmoA did not significantly affect the NMR spectra of ^15^N-labelled [C117S]YmoB. The NMR spectra of ^15^N-labelled Hha or YmoA were also not significantly affected by [C117S]YmoB. Thus, in this case, the toxin (Hha) and antitoxin (YmoB) components do not form a stable complex. However, they do interact transiently, which is enough to cause the chemical inactivation of the toxin.

This fact could be clearly demonstrated using paramagnetic relaxation enhancement NMR experiments. When a paramagnetic centre approaches, even transiently, to specific nuclei, NMR signals are strongly broadened due to fast relaxation and the corresponding decrease in signal intensity is easily measured^32^.

The native single cysteine residue of Hha was reacted with 1-oxyl-2,2,5,5-tetramethyl-3-pyrroline-3-methyl)-methanethiosulfonate (MTSL) that incorporates a nitroxide spin label. We compared the intensities of the signals from individual residues of ^15^N-labelled [C117S]YmoB in the presence and in the absence of paramagnetic Hha.

[C117S]YmoB residues perturbed by spin-labelled Hha are clustered: one of the most perturbed residues is E53 located in helix 2. Additional affected residues are located in adjacent turns of the same helix (N49, A56 and S57) and in three consecutive turns of helix 3 (E77, E78, D81 and Y84) (Fig. 5b and Supplementary Fig. 5). Of these, N49, E53, A56 and D81 are absolutely conserved in the YbaJ-superfamily. In addition, position 77 is always occupied by a negatively charged residue and position 84 by tyrosine or phenylalanine (Fig. 5d). The transient interaction of spin-labelled YmoA with [C117S]YmoB was similarly mapped and affected the same regions (Supplementary Fig. 5). The conserved E53 residue was mutated to lysine in TomB. The gene expressing [E53K]TomB or wildtype TomB were incorporated in the pCA24N-*tomB-hha* plasmid that also encodes the *hha* gene. The plasmids were incorporated into a *Δhha ΔtomB* host and the growth curves were compared. The strains with [E53K]TomB showed significantly higher growth confirming that the E53 residue is functionally important also in TomB (Supplementary Fig. 6). The better performance of the E53K variant suggests an improved antitoxin activity against Hha, although the possibility that TomB has some residual toxicity that is reduced in the E53K mutant cannot be ruled out.

### A channel provides access to C18 in [C117S]YmoB

A buried site in the interior of the four-helix bundle where the conserved C18 residue of YmoB is located is a candidate to be the protein ‘active site' responsible for the enhanced reaction of Hha with molecular oxygen. The YmoB C18 residue is located close to the Hha binding site. However, its buried location raises the question of how oxygen can reach the cysteine site.

Analysis of the structure of [C117S]YmoB using the DoGSiteScorer software^33^ identified a shallow pocket on the surface of [C117S]YmoB formed by K15, E19, F108, F111, S112, G113, I116, S117 and M120 (Fig. 5c). In some of the NMR structures K15, E19, F108 and F111 side chains define a narrow channel that connects the protein surface with the location of C18. The width of this channel is comparable to that of an oxygen molecule, and we hypothesized that it could facilitate its access to the interior of the four-helix bundle. To test this hypothesis, we mutated F111 of [C117S]YmoB to leucine and to tyrosine. The NMR spectra of the two mutants are typical of a well-folded protein and, in the case of the F111L mutant, chemical shifts are very similar to the wild type suggesting a very similar backbone structure (Supplementary Fig. 7). However, these two YmoB variants lost their capacity to enhance Hha oxidation *in vitro* (Fig. 3b), in agreement with the suggested role of phenylalanine side chains in channel formation and YmoB-enhanced oxidation of Hha.

### YmoB cysteines are not essential for YmoB antitoxin activity

YmoB or [C117S]YmoB at 200 μM concentration were incubated in the presence of air, for 1 day at room temperature with 1:10 dimedone, a specific reagent for sulfenic acid detection yielding a stable adduct with a characteristic mass increment, in the presence and in the absence of 2 mM dithiothreitol (DTT). Disulfide-bonded oligomers were separated by SEC and monomeric samples were then digested with GluC endopeptidase. Samples were analysed by liquid chromatography coupled to tandem mass spectrometry to identify peptides containing oxidized cysteine residues. All possible SO_*x*_H oxidized forms were detected (Supplementary Fig. 8). In addition, the observation of a dimedone adduct of C117 in wild-type YmoB confirmed the formation of sulfenic acid. Although sulfenic acid was also detected by the mass increment of the corresponding peptides, no adduct with C18 was observed.

In order to test whether YmoB cysteine residues are essential for the antitoxin activity, we compared *E. coli Δhha* growth for cells overexpressing Hha and wildtype YmoB, [C117S]YmoB, [C18S,C117S]YmoB or no antitoxin (Supplementary Fig. 9). Strains co-expressing Hha and the cysteine-free YmoB variant showed a longer lag time than those co-expressing Hha and YmoB. Even so, the final cell density at the stationary phase reached the same level as in the controls not expressing Hha. Thus, the presence of cysteines is not essential for the antitoxin activity of YmoB, although they contribute to make it more efficient.

## Discussion

The four-helix bundle is highly conserved in a set of 250 TomB homologues while the C-terminal region, following helix H4, shows the largest variability (Fig. 5d and Supplementary Fig. 1).

One of the most conserved regions includes cysteine 18 and its environment: position 15 is exclusively occupied by lysine or arginine, position 19 is preferentially occupied by glutamic and aspartic acid and position 17 by leucine, placing C18 in a peculiar hydrophobic but polarized environment. The hydrophobic environment is completed by aromatic residues in position 108 (phenylalanine appears in 225/250 sequences) and in position 111 (phenylalanine in 240/250 sequences) forming an access channel to C18 in [C117S]YmoB (Supplementary Fig. 10).

The residues transiently interacting with Hha and their nearest neighbours are also highly conserved: E53, N49, A56, E77, E78, D81, D82 and F84.

Thus, the TA mechanism demonstrated *in vivo* for Hha/TomB and that is operative also for the artificial Hha/YmoB pair is likely to be a general feature in the entire YbaJ-superfamily. However, in spite of the large similarities, there are distinct subfamilies that differ, for example in the number of cysteine residues. In this respect, TomB and YmoB are not the closest members of the family but, nevertheless, YmoB could functionally replace TomB as an antitoxin. We also demonstrated similar oxidation processes in YmoA, suggesting that a similar TA system may also exist in *Yersinia* sp.

A homology model of TomB was built from the structure of [C117S]YmoB using Modeller^34^. TomB has four cysteines, in positions 18, 25, 110 and 124. C110 is located at the end of helix H4, not far from the location of cysteine 117 in YmoB, and C124, located at the C-terminus of TomB that extends beyond the end of YmoB.

TomB cysteines 18 and 25 are located in helix H1 and are both buried in the interior of the four-helix bundle. Interestingly C18 and C25 are located symmetrically, but in opposite directions, with respect to the Hha interaction site. The residues flanking C18 (Leu-^18^Cys-Glu) and C25 (Asp-^25^Cys-Leu) form a symmetrical pattern. The residues surrounding C18 in the 3D structure of [C117S]YmoB and in the homology model of TomB are identical.

The conservation of the buried cysteine residues, their location close to the Hha interaction site, and the fact that C18 in YmoB experiences similar oxidation processes as those observed in Hha suggest that this residue may be involved in the oxidation enhancement mechanism. However, the fact that a variant of YmoB without any cysteine retained some capacity to act as an antitoxin indicates that the cysteines of YmoB are not essential. On the other hand, mutations affecting the channel leading to the YmoB C18 site prevented the oxidation of Hha. Thus, the environment around YmoB C18, rather that the cysteine residue itself is probably sufficient for YmoB-enhanced oxidation, although the cysteine residue may play a role, for example through its transient and reversible oxidation to sulfenic acid. TomB seems to have this site duplicated while the Hha-binding site is not. These structural variations among the YbaJ superfamily probably reflect adaptions to the required levels of antitoxin activity or their oxygen dependency.

The cysteine residues of Hha and YmoA are partially oxidized spontaneously by air *in vitro* even in the absence of TomB or YmoB giving a mixture of sulfenic, sulfinic and sulfonic acid. However, the oxidation is enhanced in the presence of [C117S]YmoB. This cysteine residue is conserved in nearly all members of the Hha family but its function was unknown. It is located in a loop between to helical regions, only partially solvent exposed, and located in the interface between regions with opposite charge^35^,^36^. The unusual sensitivity to oxidation may be related to this particular environment. Interestingly, C18 in YmoB is also located in a buried region but surrounded by charged residues.

Reactions of free molecular oxygen have a high-activation barrier associated with the symmetry of its electron spin triplet ground state^37^. The presence of polar residues, electric dipoles or interactions breaking the local symmetry enhances the capacity of molecular oxygen to oxidize cysteine to SO_*x*_H species^22^,^38^.

Hha oxidation leads to a loss of structure thus explaining its inactivation by TomB in the presence of oxygen. The Hha structure was also destabilized by a mutation introducing a negative charge at the position of C18. The sensitivity of Hha to charge modifications had been previously observed and is consistent with its unusual high density of charged residues^23^,^25^.

Overall, these results strongly suggest that the TA system formed by proteins from the Hha-YbaJ superfamily acts through a mechanism that does not fall in any of the currently accepted TA classes. While known TA systems are based on a stable toxin that is neutralized by forming a complex with a labile antitoxin, the Hha-YbaJ system is based on the chemical inactivation of the toxin caused by oxidation, enhanced in the presence of the antitoxin. This process does not require the formation of a stable TA complex but takes place through transient interactions.

The described mechanism links the TA system to the concentration of oxygen, making proteins from the YbaJ-superfamily effective antitoxins only for highly oxygenated cells. Strong oxygen concentration gradients in biofilms could cause the antitoxin to be less efficient in cells located in anoxic regions, resulting in selective death of these cells and the creation of cavities in the biofilm matrix allowing the escape of planktonic cells, or detachment of entire microcolonies facilitating biofilm dispersion.

The structure of [C117S]YmoB provides hints on the chemical origin of this activity and suggests that the members of the YbaJ-superfamily could be targets for antibacterial treatments to inhibit the formation of biofilms, one of the strongest contributors to the pressing antibiotic resistance problem.

## Methods

### Antitoxin activity of TomB, and YmoB variants

*Growth curves*. One colony of *E. coli* K-12 MG1655 *Δhha* cells harbouring pCA24N-*hha* (under the control of T5lac promoter, activated by 1 mM IPTG (isopropyl β-D-1-thiogalactopyranoside), selected with 100 μg ml^−1^ of ampicillin) and pBAD30-*tomB* or pBAD30-*ymoB* or pBAD30-*ymoB*(C117S) or pBAD30-*ymoB*(C18S)(C117S) (under the control of *araC* promoter, activated by 0.1% arabinose, selected with 50 μg ml^−1^ of chloramphenicol) was pre-cultured overnight at 37 °C and 250 r.p.m. in 30 ml of tryptone minimal medium (TMM: 10 g l^−1^ tryptone and 2.5 g l^−1^ NaCl).

Growth curves were started by adding 4 ml of preculture to 200 ml of TMM (with corresponding antibiotics and chemical inducers) and the experiments were carried out with the incubators and the room maintained at 37 °C.

*Oxygen saturation measurements*. Dissolved oxygen was measured as the percentage of saturation using a polarographic electrode (VWR DO210) using the built-in temperature and ionic-strength compensation. The electrode was cleaned with 14% ammonium hydroxide for 2 min and MilliQ water for 1 min to prevent cross-contamination.

*Colony forming unit measurements*. The number of CFUs was obtained by platting 10 μl of a 10^8^ dilution of samples taken at selected optical densities.

*Microtiter biofilm assays*. Single colonies of *E. coli* K-12 MG1655 *Δhha* cells harbouring pCA24N-*hha* and pBAD30-*tomB* or pBAD30-*ymoB* or pBAD30-*ymoB*(C117S) were inoculated into 25 ml TMM medium (with the corresponding antibiotic) and incubated overnight at 37 °C and 250 r.p.m. shaking speed. Two independent cultures were prepared for each strain, diluted with fresh TMM (with the corresponding antibiotic) to obtain an OD_600_ below 0.05, and 300 μl of culture were added into separate wells of a 96 well plate. Blanks were prepared with pure TMM medium. The plate was incubated at 37 °C without shaking, for 15 h. To record the total growth, we measured OD_620_. Supernatant was discarded and the plate was washed by dipping three times in MilliQ water, 300 μl of 0.1% crystal violet were added in each well and the plate was incubated for 20 min at room temperature. Staining solution was discarded and the plate was washed by dipping three times in MilliQ water. An amount of 300 μl of 95% ethanol was added to each well and was incubated for 5 min. We measured the total biofilm reading OD_540_ after mixing for 50 s. Standard biofilm formation was calculated as OD_540_/(OD_620_− OD_620_). OD measurements were done using Infinite M1000 PRO plate reader.

*Flow cell biofilm assays*. Strain *E. coli* K-12 MG1655 *Δhha* pCA24N-*hha* pBAD30-*ymoB* was streaked on LB agar plates with the corresponding antibiotics. One-day old single colonies were inoculated in 25 ml of TMM overnight at 37 °C at 250 r.p.m. Overnight cultures were diluted to reach OD_600_ below 0.05 in 150 ml TMM with the corresponding antibiotics. Diluted cultures under stirring at 37 °C were pumped to the flow cell at 10 ml h^−1^ for 3 h to facilitate cell attachment to the glass slide surface. Culture medium was replaced with 200 ml of fresh TMM containing the corresponding antibiotics and inducers and then fed to the flow cell at the same rate for 15 h. After incubation, the flow cell unit was disconnected and blocked (never allowing it to dry). It was placed at room temperature and the SYTO9 mix was added using a 5 ml syringe. The flow cell was incubated with SYTO9 in the dark for 20 min at room temperature. Eight images in random positions were taken with an Olympus FV100 confocal microscope using a long working distance × 40 Olympus LWDCDPLAN 40LP lens. 3D images of each spot were obtained using IMARIS software, and quantitative analysis of biofilm was performed using COMSTAT software. For the SYTO9 stain preparation, the LIVE/DEAD BacLight Bacterial Viability Kit L7007 was used. Three millilitre of dye was prepared in a 30 ml glass beaker mixing; 7.5 μl of component A (SYTO 9 dye, 1.67 mM / propidium iodide, 1.67 mM), 7.5 μl of component B (SYTO 9 dye, 1.67 mM / propidium iodide, 18.3 mM) and 3 ml of TMM.

### YmoB mutagenesis

Mutations in YmoB (cysteine to serine, phenylalanine to leucine, phenylalanine to tyrosine) were introduced using the QuickChange site directed mutagenesis Kit (Stratagene) with the appropriate complementary mutagenic primers. We confirmed the constructs by DNA sequencing.

### [C117S]YmoB 3D NMR structure.

*[C117S]YmoB sample preparation for NMR experiments.*^13^C/^15^N-labelled [C117S]YmoB variant was expressed in *E. coli* BL21 (DE3) in minimal medium with ^15^NH_4_Cl and/D-[^13^C] glucose as the sole sources of nitrogen and carbon respectively^39^. Cells were lysed by sonication. Soluble protein was purified by His-Tag affinity chromatography (Ni-NTA agarose resin QIAGEN) and samples were eluted using buffer A (20 mM Trizma hydrochloride, 800 mM NaCl, 400 mM imidazole, 5 mM DTT and pH=8.00). Protein was further purified by SEC using HiLoad Superdex 75 prepgrade columns (GE Healthcare) and buffer B (pH=7.00 buffered with 20 mM [NaH_2_PO_4_+Na_2_HPO_4_], 150 mM NaCl, 1 mM tris-carboxyethylphosphine (TCEP), 0.1 mM ethylenediaminetetraacetic acid (EDTA) and 0.01% w/v NaN_3_). Fractions containing the purified protein were concentrated using a centrifugal concentrator VIVASPIN 20 10,000 MWCO PES to the mentioned concentrations for NMR experiments.

*NMR acquisition, processing and analysis.* NMR spectra listed in Supplementary Table 2 were acquired at 1.4 mM protein concentration, in buffer A and 25 °C on 600 or 800 MHz Bruker spectrometers equipped with a cryo-pulse-field gradient triple-resonance probe. NMR spectra listed at Supplementary Table 3 were acquired at 1.0 mM protein concentration in buffer B and 25 °C on 900 or 800 MHz Agilent spectrometers equipped with a cryo- (900 MHz) and room temperature (800 MHz) pulse-field gradient triple- resonance probes. Hydrogen bonds were identified from a H/D exchange experiment. A lyophilized sample of 1.4 mM [C117S] YmoB was suspended in D_2_O reaching the same concentration, and a two-dimensional ^1^H-^15^N-heteronuclear single quantum correlation (HSQC) experiment was acquired after 4 min of sample preparation. Stereospecific assignments of valine and leucine methyl groups were obtained from a constant time ^1^H–^13^C HSQC on a 10% ^13^C/100% ^15^N [C117S]YmoB 1.4 mM sample in buffer B. One fresh sample of ^15^N–^13^C labelled [C117S]YmoB at 1.0 mM in buffer B was used for NOE correlation spectroscopy (NOESY) experiments and an equivalent fresh sample for total correlation spectroscopy (TOCSY) experiments. Protein distance restraints were obtained from 3D ^1^H–^15^N edited NOESY HSQC, 3D ^1^H–^13^C edited NOESY (aromatic optimized in D_2_O), and 3D ^1^H–^13^C edited NOESY HSQC in D_2_O experiments. NOESY and TOCSY spectra were recorded using gradient sensitivity-enhanced pulse sequences from the BioPack library (Agilent Inc.). Mixing time in NOESY experiments was set to 100 ms and to 14 ms in TOCSY experiments. The non-uniform sampling (NUS) schedules were prepared by program nussampler from the MDDNMR software package^40^. When appropriate, the sampling probability density was biased for aliphatic ^13^C-aliphatic evolution according to the single-bond ^13^C–^13^C homonuclear coupling constant of 35 Hz. When appropriate, sampling was exponentially weighted according to the maximum evolution time. Data processing and analysis were carried out with MDDNMR, NMRPipe^41^, NMRViewJ^42^ and CARA^43^. Proton chemical shifts were referenced using 4,4-dimethyl-4-silapentane-1-sulfonic acid as an internal standard, whereas ^15^N and ^13^C chemical shifts were indirectly referenced.

*Structure determination*. NOE distance restraints were obtained iteratively using the Unio'10/CYANA 2.1 (refs 27, 28, 29) suite program and manually inspected. Structure was determined by simulated annealing using the torsion angle dynamic simulation program CYANA 2.1 and further water refinement with CNS 1.2.1 (refs 30, 31). Protein structure calculation was based on Unio'10/CYANA generated upper distances using only unambiguously assigned restraints derived from NOESY experiments, hydrogen bond restraints based on H/D ^1^H-^15^N-HSQC exchange experiments and dihedral angle restraints calculated with TALOS+ (ref. 44). An ensemble of 100 protein structures was generated and the 20 lowest energy models were checked using PROCHECK-NMR^45^. None of the structures contained distance or dihedral angle violations >0.5 Å or 5°, respectively. Inter-helical angles were measured with Chimera v10.1 (ref. 46). Ramachandran analysis is as follows: 93.1% most favoured regions, 6.3% additional allowed regions, 0.5% generously allowed regions and 2.2% disallowed regions. Molecular images were generated with Chimera, Gimp (http://www.gimp.org/) and InkScape (https://inkscape.org/en/). Pocket identification was carried out with DogSiteScorer server^33^ using default settings. The TomB homology model was built using Modeller^34^ starting with YmoB as template structure and only rebuilding the regions surrounding insertions or deletions in the sequence alignment. Logo representation was generated using web service from http://weblogo.threeplusone.com^47^ and manually coloured using InkScape.

### Paramagnetic relaxation enhancement (PRE-NMR) experiments

^15^N-labelled [C117S]YmoB, Hha and YmoA proteins were prepared as previously described for ^13^C/^15^N-labelled [C117S]YmoB. For MTSL-protein functionalization, reductant was firstly removed using PD10 desalting column (GE). Commercially available MTSL tag (1-oxyl-2,2,5,5-tetramethyl-3-pyrroline-3-methyl)-methanethiosulfonate from Santa Cruz Biotechnology) was dissolved in acetone and 10 molar equivalents of MTSL were added to the protein sample and left reacting overnight at 4 °C under stirring conditions. Excess of MTSL was removed using P10 desalting column in buffer containing 20 mM [NaH_2_PO_4_+Na_2_HPO_4_] pH=7, 150 mM NaCl, 0.1 mM EDTA. Protein samples were prepared fresh and measured immediately after preparation. NMR experiments were recorded at 298 K with a 600 MHz Bruker Avance III spectrometer equipped with a TCI cryo-probe. NMR spectra were analysed using CCPNMR Analysis^48^.

### Mass spectrometry

*Hha oxidation by [C117S]YmoB*. A solution containing 500 μM Hha and 500 μM [C117S]YmoB was incubated for 30 min in 20 mM [NaH_2_PO_4_+Na_2_HPO_4_], 150 mM NaCl, 0.1 mM EDTA pH=7.00. Parallel experiments were carried out with the same batch of Hha with [F111L,C117S]YmoB, [F111Y, C117S]YmoB or without any YmoB variant. Hha and YmoB were separated by SEC in 50 mM ammonium acetate buffer. Hha was digested with trypsin and the resulting peptides were analysed by nanoHPLC-ESI-MS/MS (nano high-performance liquid chromatography coupled to tandem mass spectrometry with nanoelectrospray ionization) in a LTQ-FT Ultra (Thermo Scientific) mass spectrometer and processed and analysed with Xcalibur software (versus 2.02 SR2). Ion deconvolution to zero charged monoisotopic masses was performed using Xtract algorithm in Xcalibur software.

The relative abundance of the oxidized species was estimated from the ratio of the peptide spectrum matches (PSM) corresponding to peptides with oxidized cysteine (sulfenic, sulfinic or sulfonic acids) with respect to the PSM number of all cysteine-containing peptides. The incubation and digestion steps were carried out at least in duplicate and at least two separate samples of each peptide mixture were analysed by mass-spectrometry.

In a separate experiment the relative abundance of oxidized peptides was also calculated considering the chromatographic peak areas of the corresponding peptides using Skyline 3.1 software^49^, obtaining equivalent results (data not shown).

### Extended air oxidations of YmoB, [C117S]YmoB and YmoA

YmoB or [C117S]YmoB at 200 μM protein concentration and YmoA alone or in the presence of [C117S]YmoB were incubated in the presence of air for 12 days in a buffered solution of 20 mM [NaH_2_PO_4_+Na_2_HPO_4_] pH=7.0, 150 mM NaCl, 0.1 mM EDTA and 0.01% w/v NaN_3_) with or without DTT (2 mM) and in the presence of dimedone in a 1:10 ratio. Peptides were generated with endoproteinase GluC digestion and analysed as described above.

### Toxicity tests of Hha and TomB variants.

Overnight cultures of *E. coli* BW25113 Δ*hha* harbouring pCA24N, pCA24N-*hha*, pCA24N-*hha*(C18E) or pCA24N-*hha* (C18I) were inoculated into 25 ml of LB medium with 30 μg ml^−1^ chloramphenicol and 1 mM IPTG at an OD_600_ of 0.04. For TomB and [E53K]TomB, overnight cultures of *E. coli* ATCC25404 Δ*hha* Δ*tomB* harbouring pCA24N-*tomB*-*hha* or pCA24N-*tomB*(E53K)-*hha* were inoculated into 25 ml of LB medium with 30 μg ml^−1^ chloramphenicol at an OD_600_ of 0.05. After an hour of inoculation, 0.1 mM IPTG was added. Growth was evaluated periodically by measuring OD_600_. For each strain, two or three independent cultures were evaluated. To check total protein expression, cultures at an OD_600_ of 0.5 were induced with 1 mM IPTG for 4 h. Cells were sonicated, centrifuged, and 25 μg of cell lysates (supernatant) were resolved via 18% SDS–polyacrylamide gel electroporesis. Total protein of cell lysates was quantified using the Pierce BCA Protein Assay Kit (Thermo Scientific, Waltham, MA).

### Stability tests of [C18E]Hha and oxidized Hha

The *hha*(C18E) mutation was generated in a pET15b expression plasmid^35^ that produces a His_6_-tagged form of Hha. The ^15^N-labelled wild type and [C18E] proteins were purified by His-Tag affinity chromatography (Ni-NTA agarose resin QIAGEN) by eluting with 20 mM Trizma hydrochloride, 800 mM NaCl, 400 mM imidazole, 5 mM DTT and pH=8.00. A final purification step on a Superdex 75 column in 20 mM sodium phosphate, 150 mM NaCl, 1 mM TCEP, 0.01% w/v NaN_3_, pH=7.00 yielded pure proteins as monomers.

Before H_2_O_2_ treatment, TCEP was removed by passing the protein solution through a PD-10 column. After measuring the reference NMR spectra, the wild-type protein was treated with 5 mM H_2_O_2_ and spectra were recorded every 130 min.

### Data availability

All relevant data are available from the authors. NMR assignment and the structural models have been deposited in the RCSB PDB with accession numbers RCSB104115 and 2MN2.

## Additional information

**How to cite this article:** Marimon, O. *et al*. An oxygen-sensitive toxin–antitoxin system. *Nat. Commun.* **7,** 13634 doi: 10.1038/ncomms13634 (2016).

**Publisher's note:** Springer Nature remains neutral with regard to jurisdictional claims in published maps and institutional affiliations.

## Supplementary Material

Supplementary Information Supplementary Figures 1-10 and Supplementary Tables 1-4.

## Figures and Tables

**Table 1:** NMR constrains and refinement statistics.

NOE restrains (total)	1,349
Intra-residue	541
Inter-residue	808
Sequential	353
*Non-sequential*	455
Medium range	241
Long range	214
Hydrogen bonds	36
*Torsion angle restraints (total)*	198
ϕ	99
ψ	99
Total restraints per residue	13
Total restraints per residue in structured residues*	16
*Structure calculation statistics*	
*Violations*	
Distance constraints (Å)	0.0288±0.0024
Dihedral angle constrains (°)	0.2968±0.0453
Max. dihedral angle violations (°)	0.5
Max. distance constraint violation (Å)	5
*Average pairwise RMSD (Å) for all atoms*	
Heavy atoms (Å)	2.62±0.44
Backbone atoms (Å)	1.89±0.45
Average pairwise RMSD (Å) for atoms in structured residues^*^	
Heavy atoms (Å)	1.49±0.12
Backbone atoms (Å)	0.87±0.14
*Ramachandran analysis*	
Most favoured regions	93.1%
Additional allowed regions	6.3%
Generously allowed regions	0.5%
Disallowed regions	0.2%

*Structured Residues were considered the amino acids forming α-helices; K7-G30, A43-K65, G70-F87, D94-F111, I116- L119, a total of 87 amino acids from 122. NMR, muclear magnetic resonance; RMSD, root mean square deviation.
