# Supplementary material for: An oxygen-sensitive toxin–antitoxin system
Source: Nat Commun. 2016 Dec 8;7:13634. doi: 10.1038/ncomms13634 (PMC5155140; doi:10.1038/ncomms13634)
Supplement: Supplementary Information — Supplementary Figures 1-10 and Supplementary Tables 1-4. [file ncomms13634-s1.pdf]

Supplementary Figure 1: A non-redundant collection of 250 TomB homologs was obtained by BLAST search using as a query the TomB sequence from *E. coli* K12 strain (D64776). Truncated sequences and sequences with additional N-terminal peptide portions were removed. Column one is the NCBI accession number.

```
WP_036110039.1      MDEYSPKRHDIAQLKFLCESLYHDCLSSLEGSTQTWVNDPTSPASLQMNDLIEHIATFALNYKLKYEDDSDLIEKIDEYLDDTFMLFSSYSINAKDLQKWRRTGLNLFRCFSTVSKENPVSHSC-------
WP_017371454.1      MDEYSPKRHDIAQLRYLCESLYHDCLDSLGERKQGWVNDPTSTTNLQLNDLIEHIAAFALNYKIKHDADSKLIEQIDEYLDETFMLLSNYGINAKELQKWRKSGNQLFCSFVTASKDNPVSLSF-------
WP_042714473.1      MDEYSPKRHDIAQLKFLCESLHQECLANLEESHHGWVNDPTSAINLQLNELIEHIATFALNYKIKHDKDNKLIELIDEYLDDTFMLFSSYGINTHDLQKWRKTGSKLFRSFVNVSKANPVSHSC-------
WP_023478264.1      MDEYSPKRHDIAQLKFLCESLHQECLANLEESHHGWVNDPTSAINLQLNELIEHIATFALNYKIKHDKDNKLIELIDEYLDDTFMLFSSYGINTHDLQKWRKTGGKLFRSFDNVSKANPVSHSC-------
WP_044184760.1      MDEYSPKRHDIAQLKFLCETLYQDCLINLQESHHGWVNDPTSAVNLQLNELIEHIATFALNYKIKYNEDNNLVAQIDEYLDDTFMLFSSYGITTQDLQKWRKSGNRLFHCFANVSKANPVSHSC-------
WP_043085573.1      MDEYSPKRHDLAQLKFLCETLYHDCLVNLEENGAGWVNDPTAAINLQLNELIEHIATFALNYKIKYNEDDKLIAQIDEYLDDTFMLFSNYGINADDLQKWRKSGNRLFRCFINASRANPVSLSC-------
WP_024551957.1      MDEYSPKRHDIAQLKFLCESLYHDCLANLGESNHGWVNDPTSAVNLQLNELIEHIASFALNYKIKYEEDTVLIEQIDEYLDDTFMLFSNYGINAQDLQKWRKSGNRLFRCFVNASRAHPVSLSF-------
WP_007891922.1      MDEYSPKRHDIAQLKFLCESLYHDCLATLGENQHGWVNDPTSAVNLQLNDLIEHIASFGLNYKIKHEDDAALIEQLDEYLDDTFMLFSNYGINAQDLQKWRKSGNRLFWCFVNASKENPVSLSF-------
WP_015386942.1      MDEYSPKRHDIAQLKFLCESLYHDCLATLGENQHGWANDPTSAVNLQLNDLIEHIASFGLNYKIKHEDDAALIEQLDEYLDDTFMLFSNYGINAQDLQKWRKSGNRLFRCFVNASKENPVSLSF-------
WP_007706157.1      MDEYSPKRHDIAQLKFLCESLYHDCLATLGENQHGWVNDPTSAVNLQLNDLIEHIASFGLNYKIKHEDDTALIEQLDEYLDDTFMLFSNYGINAQDLQKWRKSGNRLFRCFINASKENPVSLSF-------
WP_004386908.1      MDEYSPKRHDIAQLKFLCESLYHDCLATLGENQHGWVNDPTSAVNLQLNDLIEHIASFGLNYKIKHEDDAALIEQLDEYLDDTFMLFSNYGINAQDLQKWRKSGNRLFRCFVNASKENPVSLSF-------
WP_007781481.1      MDEYSPKRHDIAQLKFLCESLYHDCLATLGENQHGWVNDPTSAVNLQLNDLIEHIASFGLNYKIKHEDDAALIEQLDEYLDDTFMLFSNYGINAQDLQKWRKSGNRLFRCFINASKENPVSLSF-------
WP_007673676.1      MDEYSPKRHDIAQLKFLCESLYHDCLATLGENQHGWVNDPTSAVNLQLNDLIEHIASFALNYKIKHEEDAALIEQLDEYLDDTFMLFSNYGINAQDLQKWRKSGNRLFRCFDNASKENPVSLSF-------
WP_038864291.1      MDEYSPKRHDIAQLKFLCESLYHDCLATLGENQHGWVNDPTSAVNLQLNDLIEHIASFGLNYKIKHDEDAALIEQLDEYLDDTFMLFSNYGINAQDLQKWRKSGNRLFRCFVNASRENPVSLSF-------
WP_007713750.1      MDEYSPKRHDMAQLKFLCESLYHDCLATLGENQHGWVNDPTSAVNLQLNDLIEHIASFALNYKIKHEEDAALIEQLDEYLDDTFMLFSNYGINAQDLQKWRKSGNRLFRCFVNASRENPVSLSF-------
WP_002438967.1      MDEYSPKRHDIAQLRFLCETLYHDCLANLGETNRGWVNDPTSAINLQLNDLIEHIATFALNYKIKYDDDSKLIEQIDEYLDDTFTLFSNYGIDAKDLQKWQKSGNRLFRCFVNASRANPVSLSLEK-----
WP_039032404.1      MDEYSPKRQDIAQLKFLCESLYHDCLTNLDESNHGWVNDPTSAINLQLNELIEHIATFALNYKIKYNEDNKLIEQIDEYLDDTFMLFSSYGINAQDLQKWRKSGNRLFRCFVTASRANPLASPVKTITNN-
WP_043866710.1      MDEYSPKRHDIAQLKFLCENLYHDCLITLGDSNHGWVNDPTSAINLQLNELIEHIASFALNYKIKHDEDNALIEHVDEYLDDTFMLFSNYGISAQDLQKWRKSGNRLFRCFVNVSKANPVSLSF-------
WP_002463720.1      MDEYSPKRHDIAQLKFLCENLYHDCLLTLGDSNHGWVNDPTSAINLQLNELIEHIASFALNYKIKHDEDNALIEHVDEYLDDTFMLFSNYGISAQDLQKWRKSGNRLFRCFVNVSKANPVSLSF-------
WP_024549710.1      MDEYSPKRHDIAQLKFLCETLYRDCLTNLGESNHGWVNDPTSAINLQLNELIEHIATFALNYKIKHDEDNILIEQIDEYLDDTFMLFSNYGINAQDLQKWRKSGNRLFRCFLNVSRANPVSLSF-------
WP_034458068.1      MDEYSPKRHDIAQLKFLCENLYHDCLTNLGESNHGWVNDPTSAINLQLNELIEHIAAFALNYKIKYDDESKLIEQIDEYLDDTFMLFSNYGINAQDLQKWRKSGNRLFRCFVTESRANPVSLSF-------
WP_034493745.1      MDEYSPKRHDIAQLKFLCENMYHDCLTNLGESNHGWVNDPTSAINLQLNELIEHIAAFALNYKIKYDDESKLIEQIDEYLDDTFMLFSNYGINAQDLQKWRKSGNRLFRCFVTESRANPVSLSF-------
WP_016537663.1      MDEYSPKRHDIAQLKFLCESLYHDCLTNLGESNHGWVNDPTSAINLQLNELIEHIAASALNYKIKYPDESKLIEQIDEYLDDTFMLFSNYGINAQDLQRWRKSGNRLFRCFVTESRANPVSHSF-------
WP_008460297.1      MDEYSPKRHDIAQLKFLCESLYHDCLTNLGESNHGWVNDPTSAINLQLNELIEHIAASALNYKIKYPDESKLIEQIDEYLDDTFMLFSNYGINAQDLQRWRKSGNRLFRCFISESRANPVSHSF-------
WP_004099648.1      MDEYSPKRHDIAQLKFLCETLYHDCLANLEESNHGWVNDPTSAVNLQLNELIEHIAAFALNYKIKYAEDNKLVTQLDEYLDDTFVLFSNYGINTADLQKWRKSGNRLFRCFVNASRENPASLSC-------
WP_004848337.1      MDEYSPKRHDIAQLKFLCETLYHDCLANLEESNHGWVNDPTSADNLQLNELIEHIAAFALNYKIKYAEDNKLITQIDEYLDDTFVLFSNYGINSADLQKWRKSGNRLFRCFVNASRENPVSLSC-------
WP_009653563.1      MDEYSPKRHDIAQLKFLCETLYHDCLANLEESNHGWVNDPTSAVNLQLNELIEHIAAFALNYKIKYAEDNKLITQIDEYLDDTFVLFSNYGINSADLQKWRKSGNRLFRCFVNASRENPVSLSC-------
WP_004129911.1      MDEYSPKRHDIAQLKFLCETLYHDCLANLEESNHGWVNDPTSAVNLQLNELIEHIAAFALNYKIKYAEDNKLITQIDEYLDDTFVLFSNYGINSADLQKWRKSGNRLFRCFVNASRENPASLSC-------
WP_025107145.1      MDEYSPKRHDIAQLKFLCETLYHDCLANLEESNHGWVNDPTSAVNLQLNELIEHIAAFALNYKIKYAEDNKLITQIDEYLDDTFVLFSNYGINSADLQKWRKSGNRLFRCFVNACRENPASLSC-------
WP_008805436.1      MDEYSPKRHDIAQLRFLCETLYHDCLANLEESNHGWVNDPTSAVNLQLNELIEHIATFALNYKIKYTEDNKLVAQVDEYLDDTFTLFSNYGINSTDLQKWKKSGNRLFRCFVNASRENPASLSC-------
WP_025710692.1      MDEYSPKRHDIAQLRFLCETLYHDCLANLEESNHGWVNDPTSAVNLQLNELIEHIATFALNYKIKYAEDNKLIDQVDEYLDDTFTLFSNYGINSSDLQKWKKSGNRLFRCFVNASRENPASLSC-------
WP_002892066.1      MDEYSPKRHDIAQLRFLCETLYHDCLANLEESNHGWVNDPTSAVNLQLNELIEHIATFALNYKIKYAEDNKLIAQVDEYLDDTFTLFSNYGINSTDLQKWKKSGNRLFRCFVNASRENPASLSC-------
WP_040227043.1      MDEYSPKRHDIAQLRFLCETLYHDCLANLEESNHGWVNDPTSAVNLQLNELIEHIATFALNYKIKYAEDYKLIAQVDEYLDDTFTLFSNYGINSTDLQKWKKSGNRLFRCFVNASRENPASLSC-------
WP_045381144.1      MDEYSPKRHDIAQLRFLCETLYHDCLANLEESNRGWVNDPTSAVNLQLNELIEHIATYALNYKIKYAEDNKLISQLDEYLDDTFMLFSSYGINTNDLQKWRKSGNRLFRCFVNASRENPVSLSC-------
WP_015367917.1      MDEYSPKRHDIAQLRFLCETLYHDCLANLEESNRGWVNDPTSAVNLQLNELIEHIATYALNYKIKYAEDNKLISQLDEYLDDTFMLFSSYGINTSDLQKWRKSGNRLFRCFVNASRENPASLSC-------
WP_045393536.1      MDEYSPKRHDIAQLRFLCETLYHDCLANLEESNRGWVNDPTSAVNLQLNELIEHIATYALNYKIKYAEDNKLISQLDEYLDDTFMLFSSYGINTSDLQKWRKSGNRLFRCFVNASRENPVSLSC-------
WP_004858785.1      MDEYSPKRHDIAQLRFLCETLYHDCLANLEESNHGWVNDPTSAINLQLNELIEHIATFALNYKIKYAEDNKLIAQLDEYLDDTFMLFSSYGINTADLQKWRKSGNRLFRCFVNASRENPASLSC-------
WP_041144356.1      MDEYSPKRHDIAQLRFLCETLYHDCLANLEESNHGWVNDPTSAVNLQLNELIDHIATFALNYKIKYAEDNKLIAQLDEYLDDTFMLFSSYGINSVDLQKWRKSGNRLFRCFVNASRENPASLSC-------
WP_031524358.1      MDEYSPKRHDIAQLKFLCETLYHDCLANLDESHHGWVNDPTSAINLQLNELIEHIATFALNYKIKHDEDNILIEQIDEYLDDTFMLFSNYGINAQDLQKWRKSGNRLFRCFLNVSRANPVSLSF-------
WP_024557393.1      MDEYSPKRHDIAQLKFLCESLYHDCLANLGESNHGWVNDPTSAVNLQLNELIEHIASFALNYKIKYEEDNVLIEQIDEYLDDTFMLFSNYGINAQDLQKWRKSGNRLFRCFVNASRANPVSLSF-------
WP_045261865.1      MDEYSPKRHDIAQLKFLCESLYHDCLANLEESNHGWVNDPTSAIYLQLNELIEHIATFALNYKIKYNEDNKLIEQIDEYLDDTFMLFSSYGINAQDLQKWRKSGNRLFRCFTNVSRANPVSLSC-------
WP_023324244.1      MDEYSPKRHDIAQLKFLCESLYHDCLANLEESNHGWVNDPTSAINLQLNELIEHIATFALNYKIKYNEDNKLIEQIDEYLDDTFLLFSSYGINAQDLQKWRKSGNRLFRCFTNVSRANPVSLSC-------
WP_045896220.1      MDEYSPKRHDIAQLKFLCESLYHDCLANLEESNHGWVNDPTSAINLQLNELIEHIATFALNYKIKYNEDNKLIEQIDEYLDDTFMLFSSYGINAQDLQKWRKSGNRLFRCFTNASRANPVSLSC-------
WP_006809850.1      MDEYSPKRHDIAQLKFLCESLYHDCLANLEESNHGWVNDPTSAINLQLNELIEHIATFALNYKIKYNEDNKLIEQIDEYLDDTFMLFSSYGINAQDLQKWRKSGNRLFRCFTNVSRANPVSLSC-------
```

WP_012016345.1	MDEYSPKRHDIAQLKFLCESLYHDCLANLDESNHGWVNDPTSAINLQLNELIEHIATFALNYKIKYNEDNKLIEQIDEYLDDTFMLFSSYGINTQDLQKWRKSGNRLFRCFSNVSRANPVSHSC-------
WP_006176939.1	MDEYSPKRHDIAQLKFLCESLYHDCLANLDESNHGWVNDPTSAINLQLNELIEHIATFALNYKIKYNEDNKLIEQIDEYLDDTFMLFSSYGINAQDLQKWRKSGNRLFRCFINASRANPVSLSC-------
WP_020885187.1	MDEYSPKRHDIAQLKFLCESLYHDCLANLDESNHGWVNDPTSAINLQLNELIEHIATFALNYKIKYNEDNKLIEQIDEYLDDTFMLFSSYGINAQDLQKWRKSGNRLFRCFINVSRANPVSLSC-------
WP_042716157.1	MDEYSPKRHDIAQLKFLCESLYHDCLANLDESNHGWVNDPTSAINLQLNELIEHIATFALNYKIKYNEDNKLIEQIDEYLDDTFMLFSSYGINAQDLQKWRKSGNRLFRFFVNVSRANPVSLSC-------
WP_045400722.1	MDEYSPKRHDIAQLKFLCESLYHDCLANLDESNHGWANDPTSAINLQLNELIEHIATFALNYKIKYNEDNKLIEQIDEYLDDTFMLFSSYGINAQDLQKWRKSGNRLFRCFVNVSRANPVSLSC-------
WP_008499288.1	MDEYSPKRHDIAQLKFLCESLYHDCLANLDESNHGWVNDPTSAINLQLNELIEHIATFALNYKIKYNEDNKLIEQIDEYLDDTFMLFSSYGINAQDLQKWRKSGNRLFRCFVNVSRANPVSLSC-------
WP_028012188.1	MDEYSPKRHDIAQLKFLCESLYHDCLANLDESNHGWVNDPTSAINLQLNELIEHIATFALNYKIKYNEDNKLIEQIDEYLDDTFMLFSSYGINTQDLQKWRKSGNRLFRCFVNVSRANPVSLSC-------
WP_042391644.1	MDEYSPKRHDVAQLKFLCETLYHDCLSNLEESHHGWVNDPTSAINLQLNELIEHIATFALNYKIKYNEDNKLIAQIDEYLDDTFMLFSSYGISTEDLQKWRKSGNRLFRCFVNVSKANPVSLSC-------
WP_041852464.1	MDEYSPKRHDIAQLKFLCETLYHDCLLNLEESHHGWVNDPTSAINLQLNELIEHIATFALNYKIKYNEDNKLIEQIDEYLDDTFMLFSSYGINTQDLQKWRKSGNRLFRCFVNVSKANPVSHSC-------
WP_007373584.1	MDEYSPKRHDIAQLKFLCETLYHDCLVNLEESHHGWVNDPTSAVNLQLNELIEHIATFALNYKIKYNEDNKLIEQIDEYLDDTFMLFSSYGINTHDLQKWRKTGNKLFRCFVNVSKANPVSHSC-------
WP_020456807.1	MDEYSPKRHDIAQLKFLCETLYHDCIVNLEESHHGWVNDPTSAINLQLNELIEHIATFALNYKIKYNEDNKLIELIDEYLDDTFMLFSSYGINTHDLQKWRKTGNKLFRSFVNVSKANPVSHSC-------
WP_017459991.1	MDEYSPKRHDIAQLKFLCETLYHDCLVNLEESHHGWVNDPTSAINLQLNELIEHIATFALNYKIKYNEDNKLIELIDEYLDDTFMLFSSYGINTHDLQKWRKTGNKLFRSFVNVSKANPVSHSC-------
WP_045509164.1	MDEYSPKRHDIAQLKFLCETLYHDCLVNLEESHHGWVNDPTSAINLQLNELIEHIATFALNYKIKYNEDNKLIEQIDEYLDDTFMLFSSYGINTHDLQKWRKTGNKLFRSFVNVSKANPVSHSC-------
WP_039056183.1	MDEYSPKRHDIAQLKFLCETLYHDCLVNLEESHHGWVNDPTSAINLQLNELIEHIATFALNYKIKYNEDNKLIEQIDEYLDDTFMLFSSYGINTHDLQKWRKTGNKLFRSFINVSKANPVSHSC-------
WP_013367303.1	MDEYSPKRHDIAQLKFLCETLYHDCLVNLEENRAGWVNDPTSAINLQINELIEHIATFALNYKIKYNEDNKLIAQIDEYLDDTFMLFSNYGINAEDLQKWRKSGNRLFRCFTNISRANPVSLSC-------
WP_038155031.1	MDEYSPKRHDIAQLKFLCETLYHDCLANLEESNHGWVNDPTSAVNLQLNELIEHIATFALNYKIKYNEDNKLIAQIDEYLDDTFMLFGNYGINAEDLQKWRKSGNRLFRCFTNVSRANPVSLSC-------
WP_006818850.1	MDEYSPKRHDIAQLKFLCETLYHDCLANLEESNHGWVNDPTSAGNLQLNELLEHIATFALNYKIKYNEDNKLITQIDEYLDDTFMLFSNYGINAEDLQKWRKSGNRLFRCFTNVSRANPVSLSC-------
WP_032616865.1	MDEYSPKRQDIAQLKFLCESLYHDCLSNLDESNHGWVNDPTSAINLQLNELIEHIATFALNYKIKYNEDNKLIEQIDEYLDDTFMLFSSYGINAQDLQKWRKSGNRLFRCFVNASKANPLASPVKIITNN-
WP_040076874.1	MDEYSPKRHDIAQLKFLCESLYHDCLANLDESNHGWVNDPTSAINLQLNELIEHIATFALNYKIKYNEDNKLIEQIDEYLDDTFMLFSSYGINSQDLQKWRKSGNRLFRCFVNASRANPLGSYVKTITNN-
WP_035891857.1	MDEYSPKRHDIAQLKFLCETLYHDCLVTLEESNHGWVNDPTSAVNLQLNELIEHIATFALNYKIKYDEDNKLIAQIDEYLDDTFMLFSNYGINSSDLQKWRKSGNKLFRCFVNASRANPVSLSC-------
WP_015965533.1	MDEYSPKRHDIAQLRFLCETLYHDCLANLEESNHGWVNDPTSAINLQLNELIEHIATFALNYKIKYNEDNKLIAQIDEYLDDTFMLFSNYGINSEDLQKWRKSGNKLFRCFVNASRANPVSLSC-------
WP_001396748.1	MDEYSPKRHDIAQLKFLCEILYHDCLANLEESNHGWGNDPTSAINLQLNELIEHIATFALNYKIKYNEDNKLIEQIDEYLDDTFMLFSSYGINMQDLQKWRKSGNRLFRCFVNATKENPASLSC-------
WP_039059917.1	MDEYSPKRHDIAQLKFLCETLYHDCLANLEESNHGWVNDPTSAINLQLNELIEHIATFALNYKIKYNEDNKLIEQIDEYLDDTFMLFSSYGINMQALQKWRKLGNRLFRCFVNATKENPASLSC-------
WP_000344810.1	MDEYSPKRHDIAQLKFLCETLYHDCLANLEESNYGWVNDPTSAINLQLNELIEHIATFALNYKIKYNEDNKLIEQIDEYLEDTFMLFSSYGINMQDLQKWRKSGNRLFRCFVNATKENPASLSC-------
WP_000344794.1	MDEYSPKRHDIAQLKFLCETLYHDCLANLEESNHGRVNDPTSAINLQLNELIEHIATFALNYKIKYNEDNKLIEQIDEYLDDTFMLFSSYGINMQDLQKWRKSGNRLFRCFVNATKENPASLSC-------
WP_032361280.1	MDEYSPKRHDIAQLKFLCETLYHDCLANLEESNHGWVNDPTSAINLQLNELIEHIATFALNYKIKYNEDNKLIEQIDEYLDDTFMLFSSYGINMQDLQKWRKSGNRLFRYFVNATKENPASLSC-------
WP_001667399.1	MDEYSPKRHDIAQLKFLCETLYHYCLANLEESNHGWVNDPTSAINLQLNELIEHIATFALNYKIKYNEDNKLIEQIDEYLDDTFMLFSSYGINMQDLQKWRKSGNRLFRCFVNATKENPASLSC-------
WP_001546152.1	MDEYSPKRHDIAQLKFLCETLYHDCLANLEESNHGWVNDPTSAINLQLNELIEHIATFALNYKIKYNEDNKLIEQIDEYLDDTFMLFSSYGINMQDLQKWRKSGNRLFLCFVNATKENPASLSC-------
WP_021578395.1	MDEYSPKRHDIAQLKFLCETLYHDCLANLEESNHGWVNDPTSAINLQLNELIEHIATFALNYKIKYNEVNKLIEQIDEYLDDTFMLFSSYGINMQDLQKWRKSGNRLFRCFVNATKENPASLSC-------
WP_000344795.1	MDEYSPKRHDIAQLKFLCETLYHDCLANLEESNHGWVNDPTSAINLLLNELIEHIATFALNYKIKYNEDNKLIEQIDEYLDDTFMLFSSYGINMQDLQKWRKSGNRLFRCFVNATKENPASLSC-------
WP_000344811.1	MDEYSPKRHDISQLKFLCETLYHDCLANLEESNHGWVNDPTSAINLQLNELIEHIATFALNYKIKYNEDNKLIEQIDEYLDDTFMLFSSYGINMQDLQKWRKSGNRLFRCFVNTTKENPASLSC-------
WP_001454689.1	MDEYSPKRHDIAQLKFLCETLYHDCLANLEESNHGWGNDPTSAINLQLNELIEHIATFALNYKIKYNEDNKLIEQIDEYLDDTFMLFSSYGINMQDLQKWRKSGNRLFRCFVNATKENPASLSC-------
WP_000344808.1	MDEYSPKRHDIAQLKFLCETLYHDCLANLEESNHGWVNDSTSAINLQLNELIEHIATFALNYKIKYNEDNKLIEQIDEYLDDTFMLFSSYGINMQDLQKWRKSGNRLFRCFVNATKENPASLSC-------
WP_000344809.1	MDEYSPKRHDIAQLKFLCETLYHDCLANLEESNYGWVNDPTSAINLQLNELIEHIATFALNYKIKYNEDNKLIEQIDEYLDDTFMLFSSYGINMQDLQKWRKSGNRLFRCFVNATKENPASLSC-------
WP_021523393.1	MDEYSPKRHDIAQLKFLCETLYHDCLANLEESNHGWVNAPTSAINLQLNELIEHIATFALNYKIKYNEDNKLIEQIDEYLDDTFMLFSSYGINMQDLQKWRKSGNRLFRCFVNATKENPASLSC-------
WP_000344797.1	MDEYSPKRHDIAQLKFLCETLYHDCLANLEESNHGWVNDPTSAINLQLNELIEHIATFALNYKIKYNEDNKLIEQIDEYLDDTFMLFSSYGINMQALQKWRKSGNRLFRCFVNATKENPASLSC-------
WP_000344789.1	MDEYSPKRHDIAQLKFLCEILYHDCLANLEESNHGWVNDPTSAINLQLNELIEHIATFALNYKIKYNEDNKLIEQIDEYLDDTFMLFSSYGINMQDLQKWRKSGNRLFRCFVNATKENPASLSC-------
WP_039719905.1	MDEYSPKRHDIAQLKFLCETLYHDCLANLEESNHGWVNDPTSAINLQLNELIEHIAIFALNYKIKYNEDNKLIEQIDEYLDDTFMLFSSYGINMQDLQKWRKSGNRLFRCFVNATKENPASLSC-------
WP_000344804.1	MDEYSPKRHDIAQLKFLCETLYHDCLANLEESNHGWVNDPTSAINLQLNELIEHIETFALNYKIKYNEDNKLIEQIDEYLDDTFMLFSSYGINMQDLQKWRKSGNRLFRCFVNATKENPASLSC-------
WP_032240772.1	MDEYSPKRHDIAQLKFLCETLYHDCLANLEESNHSWVNDPTSAINLQLNELIEHIATFALNYKIKYNEDNKLIEQIDEYLDDTFMLFSSYGINMQDLQKWRKSGNRLFRCFVNATKENPASLSC-------
WP_000344791.1	MDEYSPKRHDIAQLKFLCETLYHDCLAKLEESNHGWVNDPTSAINLQLNELIEHIATFALNYKIKYNEDNKLIEQIDEYLDDTFMLFSSYGINMQDLQKWRKSGNRLFRCFVNATKENPASLSC-------
WP_001734908.1	MDEYSPKRHDIAQLKFLCETLYHDCLANLEESNHGWVKDPTSAINLQLNELIEHIATFALNYKIKYNEDNKLIEQIDEYLDDTFMLFSSYGINMQDLQKWRKSGNRLFRCFVNATKENPASLSC-------
WP_000344798.1	MDEYSPKRHDIAQLKFLCETLYHDCLANLEESNHGWVNDPTSAINLQLNELIEHIATFALNYKIKYNEDNKLIEQIDEYLDDTFMLFSSYGINMQDLQKWRKSGNRLFRCFVNATKANPASLSC-------
EDU97823.1	MDEYSPKRHDIAQLKFLCETLYHDCLANLEESNHGWVNDPTSAINLQLNELIEHIATFALNYKIKYNEDNKLIEQIDEYLDDTFILFSSYGINMQDLQKWRKSGNRLFRCFVNATKENPASLSC-------
WP_028120519.1	MDEYSPKRHDIAQLKFLCETLYHDCLANLEESNHGWVNDPTSAINLQLNELIEHIATFALNYKIKYNEDNKLIEQINEYLDDTFMLFSSYGINMQDLQKWRKSGNRLFRCFVNATKENPASLSC-------
WP_044721071.1	MDEYSPKRHDIAQLKFLCETLYHDCLANLEESNHGWVNDPTSAINLQLNELIEHIATFALNYKIKYNEDNKLIEQIDEYLDDTFMLFSSYGINMQNLQKWRKSGNRLFRCFVNATKENPASLSC-------
WP_000344792.1	MDEYSPKRHDIAQLKFLCETLYHDCLANLEENNHGWVNDPTSAINLQLNELIEHIATFALNYKIKYNEDNKLIEQIDEYLDDTFMLFSSYGINMQDLQKWRKSGNRLFRCFVNATKENPASLSC-------
WP_001557978.1	MDEYSPKRHDIAQLKFLCETLYHDCLANLEESNHGWVNDPTSTINLQLNELIEHIATFALNYKIKYNEDNKLIEQIDEYLDDTFMLFSSYGINMQDLQKWRKSGNRLFRCFVNATKENPASLSC-------
WP_000344802.1	MDEYSPKRHDIAQLKFLCETLYHDCLANLEESNHGWVNDPTSAINLQLNELIEHIATFALNYKIKYNEDNKLIEQIDEYLDDTFMLFSSYGINMQDLQKWRKSGNRLFRCFVNTTKENPASLSC-------
WP_039004128.1	MDEYSPKRHDITQLKFLCETLYHDCLANLEESNHGWVNDPTSAINLQLNELIEHIATFALNYKIKYNEDNKLIEQIDEYLDDTFMLFSSYGINMQDLQKWRKSGNRLFRCFVNATKENPASLSC-------

```
WP_009422851.1      MDEYSPKRHDIAQLKFLCETLYHDCLANLEESNHGWVNDPTSAINLQLNELIEHIATFALNYKIKYNEDNKLIEQIEEYLDDTFMLFSSYGINMQDLQKWRKSGNRLFRCFVNATKENPASLSC-------
WP_001620248.1      MDEYSPKRHDIAQLKFLCETLYHDCLANLKESNHGWVNDPTSAINLQLNELIEHIATFALNYKIKYNEDNKLIEQIDEYLDDTFMLFSSYGINMQDLQKWRKSGNRLFRCFVNATKENPASLSC-------
WP_032170596.1      MDEYSPKRHDIAQLKFLCETLYHDCLANLEESNHGWVNDPTSAINLQLNELIEHIATFALNYKIKYNEDNKLIEQIDKYLDDTFMLFSSYGINMQDLQKWRKSGNRLFRCFVNATKENPASLSC-------
WP_021532618.1      MDEYSPKRHDVAQLKFLCETLYHDCLANLEESNHGWVNDPTSAINLQLNELIEHIATFALNYKIKYNEDNKLIEQIDEYLDDTFMLFSSYGINMQDLQKWRKSGNRLFRCFVNATKENPASLSC-------
WP_044726342.1      MDEYSPKRHDIAQLKFLCETLYHDCLANLEESNHGWVNDPTSAINLQLNELIEHIATFALNYKIKYNEDNKLIEQIDEYLDDTFMLFSSYGINMQDLQKWRKSGNRLFRCFVNATKENPADSSGGRSR---
WP_029395991.1      MDEYSPKRHDIAQLKFLCETLYHDCLANLEESNHGWVNDPTSAINLQLNELIEHIATFALNYKIKYNEDNKLIEQIDEYLDDTFMLFSSYGINMQDLQKWRKSGNRLFRCFVNATKENLASLSC-------
WP_023351501.1      MDEYSPKRHDIAQLKFLCETLYHDCLANLEESNHGWVNDPTSAINLQLNELIEHIATFALNYKIKYNEDNKLIEQIDEYLDDTFMLFSSYGINMQDLQKWRKSGNRLFRCFVNATKENSASLSC-------
WP_000344800.1      MDEYSPKRHDIAQLKFLCETLYHDCLANLEESNHGWVNDPTSAINLQLNELIEHIATFALNYKIKYNEDNKLIEQIDEYLDDTFMLFSSYGINMQDLQKWRKSGNRLFRCFVNATKENPASLSC-------
WP_000344801.1      MDEYSPKRHDIAQLKFLCETLYHDCLANLEESNHGWVNDPTSAINLQLNELIEHIATFALNYKIKYNEDNKLIEQIDEYLDDTFMLFSSYGINMQDLQKWRKSGNRLFRCFVNATKENPASLSCSNYYNHG
WP_000344799.1      MDEYSPKRHDIAQLKFLCETLYHDCLANLEESNHGWVNDPTSAINLQLNELIEHIATFALNYKIKYNEDNKLIEQIDEYLDDTFMLFSSYGINMQDLQKWRKSGNRLFRCFVNATKENP------------
WP_032332485.1      MDEYSPKRHDIAQLKFLCETLYHDCLANLEESNHGWVNDPTSAINLQLNELIEHIATFALNYKIKYNEDNKLIEQIDEYLDDTFMLFSSYGINMQDLQKWRKSGNRLFRCFVNATKEN-------------
WP_038425259.1      MDEYSPKRHDIAQLKFLCETLYHDCLANLEESNHGWVNDPTSAVNLQLNELIEHIATFALNYKIKYNEDNKLIAQIDEYLD-----FSSYGINTQDLQKWRKSGNRLFRCFVNATRANPVSLSC-------
WP_032948706.1      MDEYSPKRHDIAQLKFLCETLYHDCLANLEQSNHGWVNDPTSATSLQLNELIEHIATFALNYKIKYNEDNKLIAQIDEYLDDTFMLFSSYGINTQDLQKWRKSGNRLFHCFVNATRANPVSLSC-------
KGZ33153.1          MDEYSPKRHDIAQLKFLCETLYHDCLANLEQSNHGWVNDPTSATSLQLNELIEHIATFALNYKIKYNEENKLIAQIDEYLDDTFMLFSSYGINTQDLQKWRKSGNRLFRCFVNATRANPVSLSC-------
WP_003021733.1      MDEYSPKRHDIAQLKFLCETLYHDCLANLEQSNHGWVNDPTSATSLQLNELIEHIATFALNYKIKYNEDNKLIAQIDEYLDDTFMLFSSYGINTQDLQKWRKSGNRLFRCFVNATRANPVSLSC-------
KHE06706.1          MDEYSPKRHDIAQLKFLCETLYHDCLANLEQSNHGWVNDPTSATSLQLNELIEHIATFALNYKIKYNEDNKLVAQIDEYLDDTFMLFSSYGINTQDLQKWRKSGNRLFRCFVNATRANPVSLSC-------
WP_000344793.1      MDEYSPKRHDIAQLKFLCETLYHDCLANLEESNHGLVNDPTSAVNLQLNELIEHIATFALNYKIKYNEDNKLIAQIDEYLDDTFMLFSSYGINTQDLQKWRQSGNRLFRCFVNATRANPVSLSC-------
WP_039499470.1      MDEYSSKRHDIAQLKFLCETLYHDCLANLEESNHGWVNDPTSAVNLQLNELIEHIATFALNYKIKYNEDNKLIAQIDEYLDDTFMLFSSYGINTQDLQKWRKSGNRLFRCFVNATRANPVSLSC-------
WP_025840143.1      MDGYSPKRHDIAQLKFLCETLYHDCLANLEESNHGWVNDPTSAVNLQLNELIEHIATFALNYKIKYNEDNKLIAQIDEYLDDTFMLFSSYGINTQDLQKWRKSGNRLFRCFVNATRANPVSLSC-------
WP_000344787.1      MDEYPPKRHDIAQLKFLCETLYHDCLANLEESNHGWVNDPTSAVNLQLNELIEHIATFALNYKIKYNEDNKLIAQIDEYLDDTFMLFSSYGINTQDLQKWRKSGNRLFRCFVNATKANPVSLSC-------
WP_023231707.1      MDEYSPKRHDIAQLKFLCETLYHDCLANLEESNHGWVNDPTSAVNLQLNELIEHIATFALNYKIKYNEDNKLVAQIDEYLDDTFMLFSSYGINTQDLQKWRKSGNRLFRCFVNATRANPVSLSC-------
WP_000344806.1      MDEYSPKRHDIAQLKFLCETLYHDCLANLEESNHGWVNDPTSAVNLQLNELIEHIATFALNYKIKYNEDNKLIAQIDEYLDDTFMLFSSYGINTQDLQKWRKSGNRLFRCFVNATKANPVSLSC-------
WP_000344807.1      MDEYSPKRHDIAQLKFLCETLYHDCLANLEESNHGWVNDPTSAVNLQLNELIEHIATFALNYKIKYNEDNKLIAQIDEYLDDTFMLFSSYGINTQDLQKWRKSGNRLFRCFVNATRANPVSLSC-------
WP_012904868.1      MDEYSPKRHDIAQLKFLCETLYHDSLANLEESNHGWVNDPTSAINLQLNELIEHIATFALNYKIKYNEDNKLIEQLDEYLDDTFMLFSSYGINTQDLQKWRKSGNRLFRCFVTATRANPVSLSC-------
WP_046076572.1      MDEYSPKRHDIAQLKFLCETLYHDCLANLEESNHGWVNDPTSAVNLQLNELIEHIATFALNYKIKYNEDNKLIEQIDEYLDDTFMLFSSYGINTQDLQKWRKSGNSLFRCFVNATRANPVSLSC-------
ABV13797.1          MDEYSPKRHDIAQLKFLCETLYHDCLANLEESNHGWVNDPTSAINLQLNELIEHIATFALNYKIKYNEDNKLIAQIDEYLDDTFMLFSSYGINAQDLQKWRKSGNRLFRCFVNATRANPVSLSC-------
WP_046477029.1      MDEYSPKRHDIAQLKFLCEALYHDCLANLEESNHGWVNDPTSAINLQLNELIEHIATFALNYKIKYNEDNKLIEQIDEYLDDTFMLFSSYGINAQDLQKWRKSGNRLFRCFVNATRANPVSLSC-------
WP_042285841.1      MDEYSPKRHDIAQLKFLCETLYHDCLANLEESNHGWVNDPTSAINLQLNELIEHIATFALNYKIKYNEDNKLIEQIDEYLDDTFMLFSSYGINAQDLQKWQKSGNRLFRCFVNATRANPVSLSC-------
WP_000344803.1      MDEYSPKRHDIAQLKFLCETLYHDCLANLEESNHGWVNDPTSAINLQLNELIEHIATFALNYKIKYNEDNKLIEQIDEYLDDTFMLFSSYGINTQDLQKWRKSGNRLFRCFVNATRANPVSLSC-------
WP_042324455.1      MDEYSPKRHDIAQLKFLCETLYHDCLANLEESNHGWVNDPTSAINLQLNELIEHIATFALNYKIKYNEDNKLIEQIDEYLDDTFMLFSSYGINAQDLQKWRKSGNRLFRCFVNATRANPVSLSC-------
WP_029685718.1      MDEYSPKRYDVAQLRFLCESLSQEIYASINTQQFCWINDPTSPESLQINDLIEHIADVTIVYKLKHPEDEVLISQVDTFLDDTFTLLSRYTVNKKDFLYWEMSSKQLVKIFFDASAGQRASHQAFISQE--
WP_038018735.1      MDEYSPKRYDVAQLKFLCENLFDESIAILNVQQACWINDPASSENLQLHELIEHIAAFSILYKLKHSEDEQLIKQVDKYLDDTFTLFSGYTVNKQDFSFWKTSSNQLFRLFFEASPCETNGHSFIH-----
WP_025902458.1      MDEYSPKRYDVAQLKFLCENLFDESLATLNLQELCWINDPTSIENLQLHDLIEHIAAVSIIYKLKHSEDELLISQVDKYLDDTFTLFSGYTVNEQDLDYWKASSNKLFRLFFEASLCQESGQSLTH-----
WP_029990846.1      MDEYSPKRYDVAQLKFLCENLFDESLATLNLQELCWINDPTSIENLQLHDLIEHIAAVSIIYKLKHSEDELLISQVDKYLDDTFTLFSGYTVNEQDLNYWKASSNKLFRLFFEASLCQESGQSLTH-----
WP_039188525.1      MDEYTSYQHDITELKYLCNMLYNQGMDVLSDSHHGWVNDPTAVDNLQLNELNEHIANFSLTFKIKYPNASELTEILDEYLDETYSLFSNYSINEPELKKWLKAKGRILRYLAGEKTTSALNFNL-------
WP_004091740.1      MDEYTSYQHDITELKYLCNMLYNQGMDVLSDSHHGWVNDPTAADNLQLNELNEHIANFGLTFKIKYPNASELTEILDEYLDETYSLFSNYSINEPELKKWLKAKGRILRYLAGEKTTSALNFNL-------
WP_008814252.1      MDEYTSYQHDITELKYLCNMLYNQGMDVLSDSHHGWVNDPTAVDNLQLNELNEHIANFGLTFKIKYPNASELTEILDEYLDETYSLFSNYSINEPELKKWLKAKGRILRYLAGEKTTSALNFNL-------
WP_043494991.1      MDEYTSYQHDITELKYLCNMLYNQGMEVLSDSYHGWVNDPTAVANLQLNELNEHIATFGLTFKIKYPKSSELTEILDEYLDETYSLFSNYSINEPELKKWLKVKGRILRYLAGEKPASALNF---------
WP_046457234.1      MDEYTSYQHDITELKYLCNMLYNQGMEVLSDSHHGWVNDPTAVANLQLNELNEHIATFGLTFKIKYPKSSELTEILDEYLDETYSLFSNYSINEPELKKWLKAKGRILRYLAGEKPASALNF---------
WP_025799952.1      MDEYTSYQHDITELKYLCNMLYNQGMEVLSDSHHGWVNDPTAVANLQLNELNEHIATFGLTFKIKYPKSSELTEILDEYLDETYSLFSNYSINEPELKKWLKVKGRILRYLAGEKPASALNF---------
WP_024523031.1      MDEYTSYRHDIAELKYLCDSLYHQGMDVLEESHHGWVSDPTAMVNLQLNELIEHIANVALSFKIKYPQHSSLCEIVDDYLDETYALFSAYSISEQALRHWLRSKRRVAYSLAHEKRNAALHV---------
WP_005283520.1      MDEYTSYRHDIAELKYLCDSLYRQGMDVLEESHHGWVSDPTAMVNLQLNELIEHIANVALSFKIKYPQHSSLCEIVDDYLDETYALFSAYSVSEQALRHWLRSKRRVAYSLAHEKRNAALHV---------
WP_015870487.1      MDENTSYQHDISELKYLCDYLYHQGIDVLGESNHGWVSDPTAEVNLQLNELIEHIASIAQSFKIKYPRHSDLAEMLDYYLDETYALFGTYSISETALRQWLRTKRRMAYCLAHEKRNAALHV---------
WP_012847873.1      MDEYTSYQHDIAELKYLCDSLYHQGIDVLGESHHGWVSDPTAKVNLQLNELIEHIASVAQSFKIKYPHHSDLAEILDDYLDETYALFGAYSVSETALRHWLRTKRRVAYCLAHEKRNATLHV---------
WP_034163649.1      MDEYTSYQHDIAELKYLCDSLYHQGIDVLGESHHGWVSDPTAKVNLQLNELIEHIASVAQSFKIKYPRHSDLAEILDDYLDETYALFGAYSVSETALRHWLRTKRRVAYCLAHEKRNAALHV---------
WP_015461125.1      MDEYTSYQHDIAELKYLCDSLYHQGIDVLGESHHGWVSDPTAKVNLQLNELIEHIASVAQSFKIKYPHHSDLAEILDDYLDETYALFGAYSVSETALRHWLRTKRRVAYCLAHEKRNAALHV---------
WP_036953942.1      MDEYSPKKHDIAELKYLCNSLNRDAISSLQKTNTHWVNDLSSAQSISLNELVEHIAAFVWRFKIKYPKENLVISLVEEYLDETYNLFGSPVITFSEIIDWESMNQNLVAVLDDDLKCLTSKT---------
WP_036978350.1      MDEYSPKKHDIAELKYLCNSLNRDAISNLQKTNTHWINDLSSAQSISLNELVEHIAAFVWRFKIKYPKENLVISLVEEYLDETYNLFGSPVVTFSEIIDWESMNQNLVAVLDDDLKCLTSKT---------
WP_006662760.1      MDEYSPKKHDIAELKYLCNSLNRDAISSLQKTNTHWINDLSSAQSISLNELVEHIAAFVWRFKIKYPKENLVISLVEEYLDETYNLFGSPVVTFSEIIDWESMNQNLVAVLDDDLKCLTSKT---------
WP_042846890.1      MDEYSPKRHDIAELKYLCNSLNRDAILSLQKTNTHWINDLSSAQSAALNELIEHIAAFVWRFKIKYPKENLVISLIEEYLDETYDLFGSPVITLSEIIDWETMNQNLVSVLDDDLKCLTSKT---------
```

```
WP_004914480.1      MDEYSPKRHDIAELKYLCNSLNRDAILSLQKTNTHWINDLSSPQSAALNELIEHIAAFVWRFKIKYPKENLVISLIEEYLDETYDLFGSPVITLSEIIDWEAMNQSLVTVLDDDLKCLTSKT---------
WP_004905413.1      MDEYSPKRHDIAELKYLCNSLNRDAILSLQKTNTHWINDLSSPQSAALNELIEHIAAFVWRFKIKYPKENLVISLIEEYLDETYDLFGSPVITLSEIIDWEAMNHSLVAVLDDDLKCLTSKT---------
WP_010845061.1      MDEYSPKRYDIAELKYLCEKLIHEALSVLNRADSHWIHDLTSEKSLKLNELIEHIAAFVWNFKIKYTDHYDLSTLIDEYLDETYNLFGSDKISFLELTKWQRTNEHLTNILLHDLNASLSKT---------
WP_045968974.1      MDEYSPRRHDVAELKYLCENLIHEAFSVLNRTDNHWIHDLTSEKSLKLNELIEHIAAFVWSFKIKYSDNYTLSTLIDNYLDETYNLFGSDKISFLELTKWQKTNEHLTNILLHDLNAALSKI---------
WP_038198607.1      MDEYSPRRHDIVELKYLCENLIHEAFSVLNRADNHWINDLTSEKSLKLNELIEHIAAFVWSFKIKYADNYNLSTLIDKYLDETYNLFGSDKISCFELTKWQKTNEHLTNILLHDLNACLSKT---------
WP_038187569.1      MDEYSPRRHDIVELKYLCENLIHEAFSVLNRADNHWINDLTSEKSLKLNELIEHIAAFVWGFKIKYADNYNLSTLIDEYLDETYNLFGSDKISFFELTKWQKTNEHLTNILLHDLNACLRKT---------
WP_038191932.1      MDEYSPRRHDIVELKYLCENLIHEAFSVLNRADNHWINDLTSEKSLKLNELIEHIAAFVWSFKIKYADNYNLSTLIDEYLDETYNLFGSDKISFFELTKWQKTNEHLTNILLHDLNACLSKT---------
WP_038216802.1      MDEYSPRRHDIVELKYLCENLIHEAFSVLNRADNHWINDLTSEKSLKLNELIEHIAAFVWSFKIKYADNYNLSTLINEYLDETYNLFGSDKISFFELTKWQKTNEHLTNILLHDLNACLSKT---------
WP_039383003.1      MDEYSPKRHDIAQLKFLCENLYDESMATLSENHRDWVNDPASSANLQLNDFIEHIASFTMNYKIKHTEDESLISQIDEYLDDTFMLFSSYSINAKDLQRWQRSAKRLFNIFAEECAYLQ-QPSHSF-----
WP_033752682.1      MDEYSPKRHDIAQLKFLCESLFDESMATLTDSHHGWVNDPTSENNLQLNDLIEHIASFTMNYKIKHAEDEDLITQIDDYLDDTFMLFTNYGINAQDLNRWQRSARRLFNLFSEECAFLQ-QPSHSI-----
WP_017347855.1      MDEYSPKRHDIAQLKFLCESLFDESMATLTDSHHGWVNDPTSESNLQLNELIEHIASFTMNYKIKHTEDEELITQIDEYLDDTFMLFSNYGINTQDLQRWQRTGRRLFNLFIDECAFPQ-QPSHSI-----
WP_034828139.1      MDEYSPKRHDIAQLKFLCENLFDESMATLTDSHHGWVNDPTAESNLQLNDLIEHIASFTMNYKIKHVEDEALITQIDEYLDDTFMLFSSYGINTQDLQRWQRSARRLFNLFAEECAFLQ-QPSHSF-----
WP_013508152.1      MDEYSPKRHDIAQLKFLCENLFDESMATLTDSHHGWVNDPTSESNLQLNDLIEHIASFTMNYKIKHVEDTALITQIDEYLDDTFMLFSSYGINTQDLQRWQRSARRLFNLFAEECAFLQ-QPSHSF-----
WP_021185827.1      MDEYSPKRHDIAQLKFLCENLFDESMATLTDSHHGWVNDPTSESNLQLNDLIEHIASFTMNYKIKHVEDEALITQIDEYLDDTFMLFSSYGINTQDLQRWQRSARRLFNLFAEECAFLQ-QPSHSF-----
WP_021313568.1      MDEYSPKRHDIAQLKFLCENLFDESMATLTDSHHGWVNDPTSPGNLQLNDLIEHIASFTMNYKIKHVEDEALITQIDEYLDDTFMLFSSYGINTQDLQRWQRSAKRLFNLFAEECAYLQ-QPSHSF-----
WP_007886936.1      MDEYSPKRHDIAQLKYLCENLFDESMATLTDSHHGWVNDPTSESNLQLNDLIEHIASFTMNYKIKHVEDEALITQIDEYLDDTFMLFSSYGINTQDLQRWQRSAKRLFNLFAEECAYLQ-QPSHSF-----
WP_033738679.1      MDEYSPKRHDIAQLKYVCESLFDASMATLTDSHHGWVNDPTADGNLQLNDLMEHIASFTMNYKIKHAEDEALITQIDEYLDDTFMLFSNYSVNTQDLQRWQRSAKRLFNLFQEECAYLQ-QPSHSL-----
WP_006117939.1      MDEYSPKRHDIAQLKYVCESLFDASMATLTDSHHGWVNDPTADGNLQLNDLIEHIASFTMNYKIKHAEDEALITQIDEYLDDTFMLFSSYSVNTQDLQRWQRSAKRLFNLFQEECAYLQ-QPSHSL-----
WP_039338473.1      MDEYSPKRHDIAQLKYVCESLFDASMATLTDSHHGWVNDPTADGNLQLNDLIEHIASFTMNYKIKHAEDEALITQIDEYLDDTFMLFSNYSVNTQDLQRWQRSAKRLFNLFQEECAYLQ-QPSHSL-----
WP_028723978.1      MDEYSPKRHDIAQLKYLCESLFDESMATLTDSHHGWVNDPTAESNLQLNDLIEHIASFTMNYKIKHVEDEALITQIDEYLDDTFMLFSNYSVNTQDLQRWQRSAKRLFNVFTEECAFLQ-QPSHSL-----
WP_014606431.1      MDEYSPKRHDIAQLKYLCESLFDDSMATLTDSHHGWVNDPTAESNLQLNDLIEHIASFTMNYKIKHVEDEALITQIDEYLDDTFMLFSNYSVNTQDLQRWQRSAKRLFNVFTEECAFLQ-QPSHSL-----
WP_015700827.1      MDEYSPKRHDIAQLKYLCENLFDDSMATLTDSHHGWVNDPTAESNLQLNDLIEHIASFTMNYKIKHVEDEALITQIDEYLDDTFMLFSNYSVNTQDLQRWQRSAKRLFNVFTEECAFLQ-QPSHSL-----
WP_031374658.1      MDEYSPKRHDIAQLKYLCESLFDESMATLTDSHHGWVNDPTSESNLQLNDLIEHIASFTMNYKIKHVEDEALISQIDEYLDDTFMLFSNYGVNTQDLQRWQRSAKRLFNLFTEECAFLQ-QPSHSL-----
WP_013357010.1      MDEYSPKRHDIAQLKYLCESLFDDSMATLTDSHHGWVNDPTSESNLQLNDLIEHIASFTMNYKIKHVEDEALISQIDEYLDDTFMLFSNYGINSPDLQRWQRSAKRLFNLFTEECAFLQ-QPSHSL-----
WP_003850455.1      MDEYSPKRHDIAQLKYLCESLFDDSMATLTDSHHGWVNDPTSESNLQLNDLIEHIASFTMNYKIKHVEDEALISQIDEYLDDTFMLFSNYGVNSPDLQRWQRSAKRLFNLFTEECAFLQ-QPSHSL-----
WP_046101364.1      MDEYSPKRHDIAQLKYLCESLFDDSMATLTDSHHGWVNDPTSESNLHLNDLIEHIASFTMNYKIKHVEDEALISQIDEYLDDTFMLFSNYGINNPDLQRWQRSAKRLFNLFTEECAFLQ-QPSHSL-----
WP_009091672.1      MDEYSPKRHDIAQLKYLCESLFDDSMATLTDSHHGWVNDPTSESNLHLNDLIEHIASFTMNYKIKHVEDEALISQIDEYLDDTFMLFSNYGINNPDLLRWQRSAKRLFNLFTEECAFLQ-QPSHSL-----
WP_034912812.1      MDEYSPKRHDIAQLKFLCESLYDESLATIGDSHHGWVNDPTSAINLQINDLIEHIAAFTMVYKIKHTEDDELITQVDEYLDDTFMLFSNYGINVQDLQRWQKSVKRLFNIFAKECAPT-IQTSHSF-----
WP_023654247.1      MDEYSPRRHDIAQLKFLCENLYDEGLATLGDSHHGWVNDPTSAFNLQLNELIEHIAAFTMNYKIKHVEDADLISQIDEFMDDTFMLFSNYGINIQDLQRWQKSAKLLFNIFAKECVMSQNQASHSF-----
WP_012442200.1      MDEYSPRRHDIAQLKFLCENLYDEGLATLGDSHHGWVNDPTSTFNLQLNELIEHIAAFTMNYKIKHVEDADLISQIDEYMDDTFMLFSNYGINIQDLQRWQKSAKLLFNIFAKECVMSQNQASHSF-----
WP_012668861.1      MDEYSPKRHDIAQLKFLCENLYDESLATLGDSHHGWVNDPTSTSNLQLNDLIEHIAAFTMNYKIKHIEDCDLISQIDEYLDDTFMLFSNYGVNTHDLQRWQKSAKRLFNIFAKECLMSQVQSSHSF-----
WP_033477124.1      MDEYSPKRHDIAQLKFLCENLYDESLATLGDSHHGWVNDPTSTSNLQLNDLIEHIAAFTMNYKIKHIEDSDLISQIDEYLDDTFMLFSNYGVNNHDLQRWQKSAKRLFNIFAKECVMSQIQSSHSFSSP--
WP_013035913.1      MDEYSPKRHDIAQLKFLCENLYDESLATLGDSHHGWVNDPTSTSNLQLNDLIEHIAAFTMNYKIKHIEDSDLISQIDEYLDDTFMLFSNYGVNNLDLQRWQKSAKRLFNIFAKECVMSQIQSSHSFSSP--
WP_038630095.1      MDEYSPKRHDIAQLNFLCENLYDESMATLGDSHHGWVNDPTSATNIQLNELIEHIASFTMNYKIKHAEDEDLIVQIDDYLDDTFMLFSNYGINVQDLQRWQRSARRLFNLFAEECAQLPLQPSHSF-----
WP_017800957.1      MDEYSPKRHDIAQLKFLCESLYDESMSTLGDSHHGWVNDPTSAVNLQLNELIEHIASFTMNYKIKHVEDADLIAQIDEYLDDTFMLFSNYGINAQDLQRWQKSAKRLFNIFAEECAQRPLHASHSV-----
WP_016191643.1      MDEYSPKRHDIAQLKFLCENLYDESMTTIGDSHHGWINDPTSASNLQLNDLIEHIASFTMNYKIKHIEDSDLISQIDEYLDDTFMLFSNYGINVQELQRWRKSAEGLFSIFAEECAQVANPASHSF-----
WP_024967287.1      MDEYSPKRHDIAQLKFLCENLYDESMATLGDSHHGWVNDPTSAVNLQLNDLIEHIAAFTMNYKIKHIEDADLISLIDEYLDDTFMLFSNYGINVQDMQRWQKSAKRLFKLFAEECALVIAPASHSF-----
WP_034933441.1      MDEYSPKRHDIAQLKFLCENLYDESMATLGDSHHGWVNDPTSAINLQLNDLIEHIAAFTMNYKIKHIEDSDLISQIDEYLDDTFMLFSNYGINVQDLQRWRKSAKRLFHIFAEECAHAVVQTSHSF-----
WP_034889661.1      MDEYSPKRHDIAQLKFLCENLYDESLATIGDSHHGWVNDPTSAANLQLNELIEHIAGFTMNYKIKHIEDSDLISQIDEYLDDTFMLFSNYGINVQDLQRWQKSAKRLFSIFAEECALVPVQTSHSF-----
WP_013201058.1      MDEYSPKRHDIAQLKFVCENLYDESLATLGDSHHGWVNDPTAAVNLQLNDLIEHIAAFTMNYKIKHLEDSDLISQIDEYLDDTFMLFSNYGINVQDLQRWQKSAKRLFNIFAEECALVPVQTSHSF-----
WP_025246875.1      MDEYSPKRYDIAQLKYLCENLYDEGIASLGDSYHGWVNDPSSAVNLQLNELIEHIAANIVIFKLKYHNESELTDQVETFLDDTFTLFSSYGINNYDIQRWRRSRRRLFGTFSETELCTT-----------
WP_011410545.1      MDEYSPKRYDIAQLKYLCENLYDEGIASLGNSYHGWVNDPSSAVNLQLNELIEHIAANIVIFKLKYHNESELTDQAETFLDDTFTLFSSYGINNYDIQRWRRSRRRLFGTFSETELCTT-----------
WP_009113999.1      MDEYTPKHYDIAQLRFLCENLCDESVATLGDSSHCWVNDPTSAINLQLNELIEHIATFILTFKIKYPNESELSQQVEQYLDDTYVLFSNYGINDAELRGWQKSKKRLFGMFSEGNICTPAKT---------
WP_039346862.1      MDEYTPKHYDIAQLRFLCENLCYESIATLGDSSHGWVNDPTSAINLQLNELIEHIATFILTFKIKYPNESELSEQVEKYLDDTYVLFSNYGINDTELRRWQKSKAKLFGMFSGEDICTPAKT---------
WP_039487867.1      MDEYTPKHYDIAQLRFLCENLCDESIATLGDSSHGWVNDPTSAINLQLNELIEHIATFILTFKIKYPNESELSEQVEKYLDDTYVLFSNYGINDAELRHWQKSKAKLFGMFSGENVCTPAKT---------
WP_005976089.1      MDEYTPKHYDIAQLRFLCENLCDESIATLGDSSHGWVNDPTSAINLQLNELIEHIATFILTFKIKYPNESELSEQVEKYLDDTYVLFSNYGINDAELRRWQKSKAKLFGMFSGENVCTPAKT---------
WP_039274613.1      MDEYTPKHYDIAQLRFLCENLCDESIATLGDSSHGWVNDPTSAINLQLNELIEHIATFILTFKIKYPNESELSEQVEKYLDDTYVLFSNYGINDAELRRWQKSKAKLFGMFSGESICTPAKT---------
WP_010282247.1      MDEYTPKHYDIAQLRFLCENLCDESIATLGDSSHGWVNDPTSAINLQLNELIEHIATFILTFKIKYPNESELSEQVEKYLDDTYVLFSNYGINDAELRRWQKSKAKLFGMFSGENICTPAKT---------
WP_012764631.1      MDEYTPRNYDIAQLRFLCENLRDESIANLGDSSHGWVNDPTSAINLQLNELIEHIAAFIVTYKIKYPNEEELCERVEKYLDDTYILFSNYGINDAELSRWQKSKSELFGLFSEKSICTVVRT---------
WP_026738849.1      MDEYSPKHYDIAQLRFLCENLRDESIATLGDSNHGWVNDPTSAINLQLNELIEHIAAFIGTYKIKYPDDEELYMRVEKYLDDTYILFSNYGINDAELRRWQKSKSQLFRMLSEKSSYATVRT---------
```

```
WP_021014991.1      MDEYSPKHYDIAQLRFLCENLRDESIATLGDSSHGWVNDPTSAVNLQLNELIEHIAAFIVTYKIKYPNDEELCERVEKYLDDTYILFSNYGINDGELRRWQKSRSQLFRMFSEKSICTAVKT---------
WP_035340556.1      MDEYTPQHYDIAQLRFLCENLHDESIATLGDSSHGWVNDPTSAVNLQLNELIEHIAAFVVTYKIKYPHESGLCERVEKYLDDTYILFSNYGINDTELQKWQKSKSQLFRMFSEKNVCTVVKT---------
WP_042869322.1      MDEYTPQHYDIAQLRFLCENLHDESIATLGDSNRGWVNDPTSAVNLQLNELIEHIAAFVVTYKIKYPHEAALCERVEKYLDDTYILFSNYGINDAELRKWQKSKSQLFRMFSEKSICTVVKT---------
WP_038922044.1      MDEYTPQHYDIAQLRFLCENLHDESIATLGDSSRGWVNDPTSAVNLQLNELIEHIAAFVVTYKIKYPHEAALCERVEKYLDDTYILFSNYGINDTELRKWQKSRSQLFRMFSEKSICTVVKT---------
WP_022632575.1      MDEYTPQHYDIAQLRFLCENLHDESIATLGDSSHGWVNDPTSAVNLQLNELIEHIAAFVVTYKIKYPHEAALCERVEKYLDDTYILFSNYGINDTELRKWQKSKSQLFRMFSEKSICTVVKT---------
WP_013316870.1      MDEYTPQHYDIAQLRFLCENLHDESIATLGDSSRGWVNDPTSAVNLQLNELIEHIAAFVVTYKIKYPHEAALCERVEKYLDDTYILFSNYGINDTELRKWQKSKSQLFRMFSEKSICTVVKT---------
WP_024104994.1      MDEYTPQHYDIAQLRFLCENLHDESIATLGDSSRSWVNDPTSAVNLQLNELIEHIAAFVVTYKIKYPHEAALCERVEKYLDDTYILFSNYGINDAELQKWRKSKSQLFRMFSEKSICTVVKT---------
WP_038661190.1      MDEYTPQHYDIAQLRFLCENLHDESIATLGDSSRGWVNDPTSAVNLQLNELIEHIAAFVVTYKIKYPHEAALCERVEKYLDDTYILFSNYGINDSELQKWQKSKSQLFRMFSEKSICTVVKT---------
WP_016942061.1      MDEYTPQHYDIAQLRFLCENLHDESIATLGDSRRGWVNDPTSAVNLQLNELIEHIAAFVVTYKIKYPHEAALCERVEKYLDDTYILFSNYGINDSELQKWKKSKSQLFRMFSEKSVCTVVKT---------
WP_012883774.1      MDEYTPQHYDIAQLRFLCENLHDESIATLGDSSRGWVNDPTSAVNLQLNELIEHIAAFVVTYKIKYPHEAALCERVEKYLDDTYILFSNYGINDSELQKWKKSKSQLFRMFSEKSVCTVVKT---------
WP_042859087.1      MDEYTPQHYDIAQLRFLCENLHDESIATLGDSSRGWVNDPTSAVNLQLNELIEHIAAFVVTYKIKYPHEAALCERVEKYLDDTYILFSNYGINDSELQKWKKSKSQLFRMFSEKSICTVVKT---------
WP_033570182.1      MDEYTPQHYDIAQLRFLCENLHDESIATLGDSSRGWVNDPTSAVNLQLNELIEHIAAFVVTYKIKYPHEAALCERVEKYLDDTYILFSNYGINDAELQKWKKSKSQLFRMFSEKSSCTVVKT---------
WP_027712818.1      MDEYTPQHYDIAQLRFLCENLHDESIATLGDSSHGWVNDPTSAVNLQLNELIEHIAAFVVTYKIKYPHEAALCERVEKYLDDTYILFSNYGINDAELQKWKKSKSQLFRMFSEKSNCTVVKT---------
WP_038918199.1      MDEYTPQHYDIAQLRFLCENLHDESIATLGDSSHGWVNDPTSAVNLQLNELIEHIAAFVVTYKIKYPHEAALCERVEKYLDDTYILFSNYGINDSELQKWKKSKSQLFRMFSEKSICTVVKT---------
WP_012770725.1      MDEYTPQHYDIAQLRFLCENLHDESIATLGDSSHGWVNDPTSAVNLQLNELIEHIAAFVVTYKIKYPHEAALCERVEKYLDDTYILFSNYGINDAELQKWKKSKSQLFRMFSEKSICTVVKT---------
WP_023488989.1      MDEYSTKQHDIAQLKFLCESLYDQGIATLGDSQRGWVNDPTSSDNLQLNDLIEHIASFVISFKIKYMDENDLSALVEEYLDDTYTVFSSFSINNADLHRWQETKMRTFRMLSGESICTTIKT---------
WP_004718732.1      MDEYSPKRHDIAQLKFLCENLYDEGIATLGDSHHGWVNDPTSAVNLQLNDLIEHIASFVMSFKIKYADEIDLCELVEEYLDDTYTLFSSYGINDPDLRRWQKTKDRLFRLFSGEYLCALMKN---------
WP_025382853.1      MDEYSPKRHDIAQLKFLCESLYDEGIATLGDSHHGWINDPTSAINLQLNDLIEHIASFVMSFKIKYPDGSELSELVEEYLDDTYTLFSSYGINDPELRRWQKTKEHLFRLFSGEYVCTLMKT---------
WP_033847808.1      MDEYSPKRHDIAQLKFLCESLYDEGIATLGDSHHGWINDPTSAINLQLNDLIEHIASFVMSFKIKYPDGSQLSELVEEYLDDTYTLFSSYGINDPELRRWQKTKEHLFRIFSGEYVCTLMKT---------
WP_025475995.1      MDEYSPKRHDIAQLKFLCESLYDEGIATLGDSHHGWINDPTSAINLQLNDLIEHIASFVMSFKIKYPDGSQLSELVEEYLDDTYTLFSSYGINDPELRRWQKTKEHLFRLFSGEYV---------------
WP_038928625.1      MDEYSPKRHDIAQLKFLCESLYDEGIATLGDSHHGWINDPTSAINLQLNDLIEHIASFVMSFKIKYPDGSQLSELVEEYLDDTYTLFSSYGINDPELRRWQKTKEHLFRLFSGEY----------------
WP_002218472.1      MDEYSPKRHDIAQLKFLCESLYDEGIATLGDSHHGWINDPTSAINLQLNDLIEHIASFVMSFKIKYPDGSQLSELVEEYLDDTYTLFSSYGINDPELRRWQKTKEHLFRLFSGEYVCTLMKT---------
ERP85166.1          MDEYSPKRHDIAQLKFLCESLYDEGIATLGDSHHGWINDPTSAINLQLNDLIEHIASFVMSFKIKYPDGSQLSELVEEYLDDTYTLFSSYGINDPELRRWQKTKEHLFRLFSGEYVCT-------------
WP_005163463.1      MDEYSPKRHDVAQLKFLCESLYDEGIATLGDSHHGWVNDPTSAVNLQLNDLIEHIASFVMSFKIKYPDDGDLSELVEEYLDDTYTLFSSYGINDPELQRWQKTKERLFRLFSGEYICTLMKT---------
WP_005167670.1      MDEYSPKRHDVAQLKFLCESLYDEGIATLGDSHHGWVNDPTSAVNLQLNDLIEHIASFVMSFKIKYPDGGDLSELVEEYLDDTYTLFSSYGINDPELQRWQKTKERLFRLFSGEYICTLMKT---------
WP_032816856.1      MDEYSPKRHDIAQLKFLCESLYDEGIATLGDSHHGWVNDPTSAVNLQLNDLIEHIASFVMSFKIKYPDGGELSELVEEYLDDTYTLFSSYGINDPELHRWQKTKERLFRLFSGEYICTLMKT---------
WP_004703332.1      MDEYSPKRHDIAQLKFLCENLYDEGIATLGDSHHGWVNDPTSAVNLQLNDLIEHIASFVMSFKIKYPDDGDLSELVEEYLDDTYTLFSSYGINDPELHRWQKTKERLFRLFSGEYICTLMKT---------
CFQ44634.1          MDEYSPKRHDIAQLKFLCESLYDEGIATLGDSHHGWVNDPTAAVNLQLNDLIEHIASFVMSFKIKYPDDGDLSELVEEYLDDTYTLFSSYGINDPELCRWQKTKERLFRLFSGEYICTLMKT---------
WP_038631539.1      MDEYSPKRHDIAQLKFLCESLYDEGIATLGDSHHGWVNDPTSAVNLQLNDLIEHIASFVMSFKIKYPDGADLSELVEEYLDDTYTLFSSYGINDPELRRWQKTKERLFRLFSGEYICTLMKT---------
CFR14245.1          MDEYSPKRHDIAQLKFLCESLYDEGIATLGDSHHGWVNDPTAAVNLQLNDLIEHIASFVMSFKIKYPDDGDLSELVEKYLDDTYTLFSSYGINDPELRRWQKTKERLFRLFSGEYICTLMKS---------
WP_004873431.1      MDEYSPKRHDIAQLKFLCESLYDEGIATLGDSHHGWVNDPTAAVNLQLNDLIEHIASFVMSFKIKYPDDGDFSELVEEYLDDTYTLFSSYGINDPELRRWQKTKERLFRLFSGEYICTLMKT---------
WP_004392643.1      MDEYSPKRHDIAQLKFLCESLYDEGIATLGDSHHGWVNDPTSAVNLQLNDLIEHIASFVMSFKIKYPDGGDLSELVEEYLDDTYTLFSSYGINDPELRRWQKTKERLFRLFSGEYICTLMKT---------
WP_019212878.1      MDEYSPKRHDIAQLKFLCESLYDEGIATLGDSHHGWVNDPTAAVNLQLNDLIEHIASFVMSFKIKYPDDGDLSELVEEYLDDTYTLFSSYGINDPELRRWQKTKERLFRLFSGEYICTLMKS---------
WP_005272706.1      MDEYSPKRHDIAQLKFLCESLYDEGIATLGDSHHGWVNDPTAAVNLQLNDLIEHIASFVMSFKIKYPDDGDLSELVEEYLDDTYTLFSSYGINDPELRRWQKTKERLFRLFSGEYICTLMKT---------
WP_005182270.1      MDEYSPKRHDIAQLKFLCESLYDEGIATLGDSHHGWVNDPTSAVNLQLNDLIEHIASFVMSFKIKYPDDGDLSELVEEYLDDTYTLFSSYGINDPELRRWQKTKERLFRLFSGEYTCTLMKT---------
AHM72673.1          MDEYSPKRHDIAQLKFLCESLYDEGIATLGDSHHGWVNDPTSAVNLQLNDLIEHIASFVMSFKIKYPDDGDLSELVEEYLDDTYTLFSSYGINDPELRRWQKTKERLFRLFSGEYICTLMKT---------
WP_015698304.1      MDEYSPKRHDIAQLRYLNETLYDEGIATLGDSHHGWVNDPTSAVNLQLNELIEHIASFVMSFKIKYPDDNHLSDLVEEYLDDTYTLFSNYGINDSDLRQWQKTKKRLFRMFSGDYICTLMKT---------
WP_013576616.1      MDEYSPKRHDIAQLRYLNEMLYDEGIATLGDSHHGWVNDPTSAVNLQLNELIEHIASFVMSFKIKYPDDNHLSDLVEEYLDDTYTLFSNYGINDSDLRQWQKTKKRLFRMFSGDYICTLMKT---------
WP_034792338.1      MDEYSPKRHDIAQLRFLNESLYDEGIATLGDSHHGWVNDPTSAVNLQLNELIEHIASFVMSFKIKYPDDSPLTDLVEEYLDDTYTLFSNYGINDSDLRQWQKTKKRLFRMFSGEYVCTLMKT---------
WP_017492280.1      MDEYSPKRHDIAQLRFLNESLYDEGIATLGDSHHGWVNDPTSAVNLQLNDLIEHIASFVMSFKIKYPDETNLSDLVEEYLDDTYTLFSNYGINDSDLRQWQKTKKRLFRMFSGDYVCTLMKT---------
WP_045046077.1      MDEYSPKRHDIAQLRFLNENLYDEGIATLGDSHHGWVNDPTSAVNLQLNDLIEHIASFVMSFKIKYPDETNLSDLVEEYLDDTYTLFSNYGINDSDLRQWQKTKKRLFRMFSGDYVCALMKT---------
WP_009639080.1      MDEYSPKRHDIAQLRFLNENLYDEGIATLGDSHHGWVNDPTSAVNLQLNDLIEHIASFIMSFKIKYPDETNLSDLVEEYLDDTYTLFSNYGINDSDLRQWQKTKKRLFRMFSGDYVCALMKT---------
WP_040266826.1      MDEYSSKRHDIAQLKFLCENLYDEGIATLDDSYHGWVNDPTSFVNLQLNELIEHIASFVMSYKIKYMDESDLSELVEEYLDDAYMLFSSYGINDSDLRRWQKTKAKLFRMFSGEGICTTMKT---------
CDG47204.1          MDEYSSKRHDIAQLKFLCENLYDEGIDTLDDSYHVRVNDPTSFVNLQLNELIEHIASFVMSYKIKYMDESDLSELVEEYLDDAYMLFSSYGINDSDLRRWQKTKAKLFRMFSGEGICTTMKT---------
WP_024913900.1      MDEYSPKRHDIVQLKFLCENLYDEGMATLGDSNHGWVNDPTSSVNLQLNELIEHIASFVMSFKIKYMGESDLSELVEEYLDDTYTLFSNYGINDSDLRRWQKTRARLFGMFSGEGIYTTMKT---------
WP_015671221.1      MDEYSPKRHDIAQLKFLCENLYDEGIATLGDSHHGWVNDPTSAVNLQLNELIEHIASFVMSYKIKYMDESDFSELVEEYLDDTYTLFSNYGINDSDLRRWQKTKRRLFGMFSGEVCCTKMKT---------
WP_037394581.1      MDEYSPKRHDIAQLKFLCENLSDEGMLTLGDSNHGWVNDPTSSVNLQLNELIEHIASFVMSFRIKYMDESDLSELVEEYLDDTYTLFSSYGINDSDLRRWQKTKARLFRMFSGEGVCTTMKN---------
WP_025120365.1      MDEYSPKRHDIAQLKFLCENLYDEGIATLGDSHHGWVNDPTSAVNLQLNELIEHIASFVMSFKIKYMDESDLSELVEEYLDDTYTLFSSYGINDSDLRRWQKTKARTFRLFSGEGVCTTMKT---------
WP_021180141.1      MDEYSPKRHDIAQLKFLCENLYDEGIATLGDSHHGWVNDPTSAVNLQLNELIEHIASFVMSFKIKYMDEGDLSELVEEYLDDTYTLFSSYGINDSDLRRWQKTKARTFRLFSGEGVCTTMKT---------
WP_004954559.1      MDEYSPKRHDIAQLKFLCETLYDEGIATLGDSHHGWVNDPTSAVNLQLNELIEHIASFIMSYKIKYMDESDFSELVEEYLDDTYTLFSSYGINDSDLRRWQKTKARLFRMFSGEGICTTMKT---------
```

```
WP_038874971.1      MDEYSPKRHDIAQLKFLCENLYDEGIATLGDSHHGWVNDPTSSVNLQLNELIEHIASFVMSYKIKYIDESDLSELVEEYLDDTYTLFSSYGINDSDLRRWQKTKARLFRMFSGEDICTTMKT---------
WP_006322262.1      MDEYSPKRHDIAQLKFLCENLYDEGIATLGDSHHGWVNDPTSSVNLQLNELIEHIASFVMSYKIKYMDESDLSELVEEYLDDTYTLFSSYGINDSDLRRWQKTKARLFRMFSGEGICTTMRT---------
WP_004940312.1      MDEYSPKRHDIAQLKFLCENLYDEGIATLGDSHHGWVNDPTSSVNLQLNELIEHIASFVMSYKIKYMDESDLSELVEEYLDDTYTLFSSYGINDSDLRRWQKTKARLFRMFSGEDICTTMKT---------
WP_020825515.1      MDEYSPKRHDIAQLKFLCENLYDEGIATLGDSHHGWVNDPTSSVNLQLNELIEHIASFVMSYKIKYMDESDLSELVEEYLDDTYTLFSSYGINDSDLRRWQKTKARLFRMFSGEGICTTMKS---------
WP_044548720.1      MDEYSPKRHDIAQLKFLCENLYDEGIATLGDSHHGWVNDPTSSVNLQLNELIEHIASFVMSYKIKYMDESDLSELVEEYLDDTYTLFSSYGINDSDLRRWQKTKARLFRMFSGEGVCTTMKT---------
WP_004949348.1      MDEYSPKRHDIAQLKFLCENLYDEGIATLGDSHHGWVNDPTSSVNLQLNELIEHIASFVMSYKIKYMDESDLSELVEEYLDDTYTLFSSYGINDSDLRRWQKTKARLFRMFSGEGICTTMKT---------
```

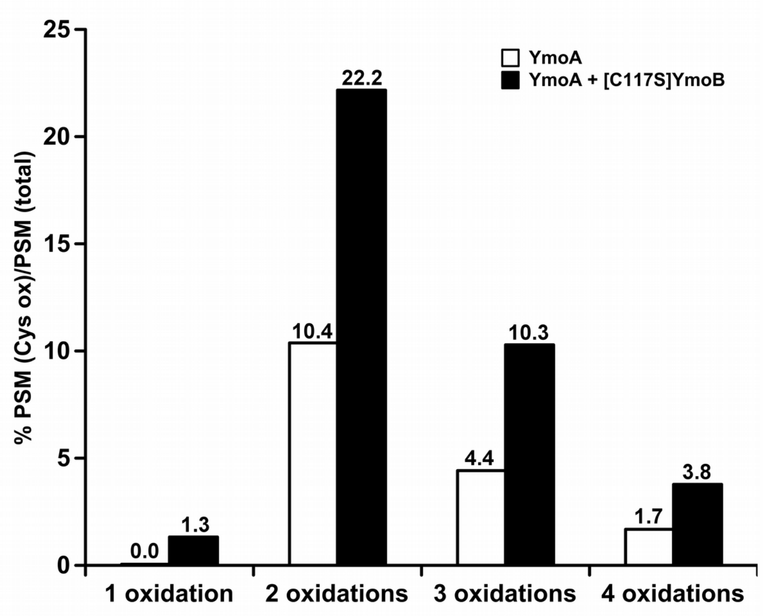

Supplementary Figure 2. Relative abundance of oxidized peptides from YmoA (600 µM) in the presence and in the absence of equimolar concentration of [C117S]YmoB. Samples were digested with GluC endopeptidase prior to ESI-MS/MS analysis. The four categories represent the number of additional oxygen atoms relative to the non-oxidized form, the forth oxidation accounts for the presence of a Methionine residue. The peptide spectrum matches (PSM) of all peptides in the same oxidation level were pooled and compared with the PSM number for all detected peptides.

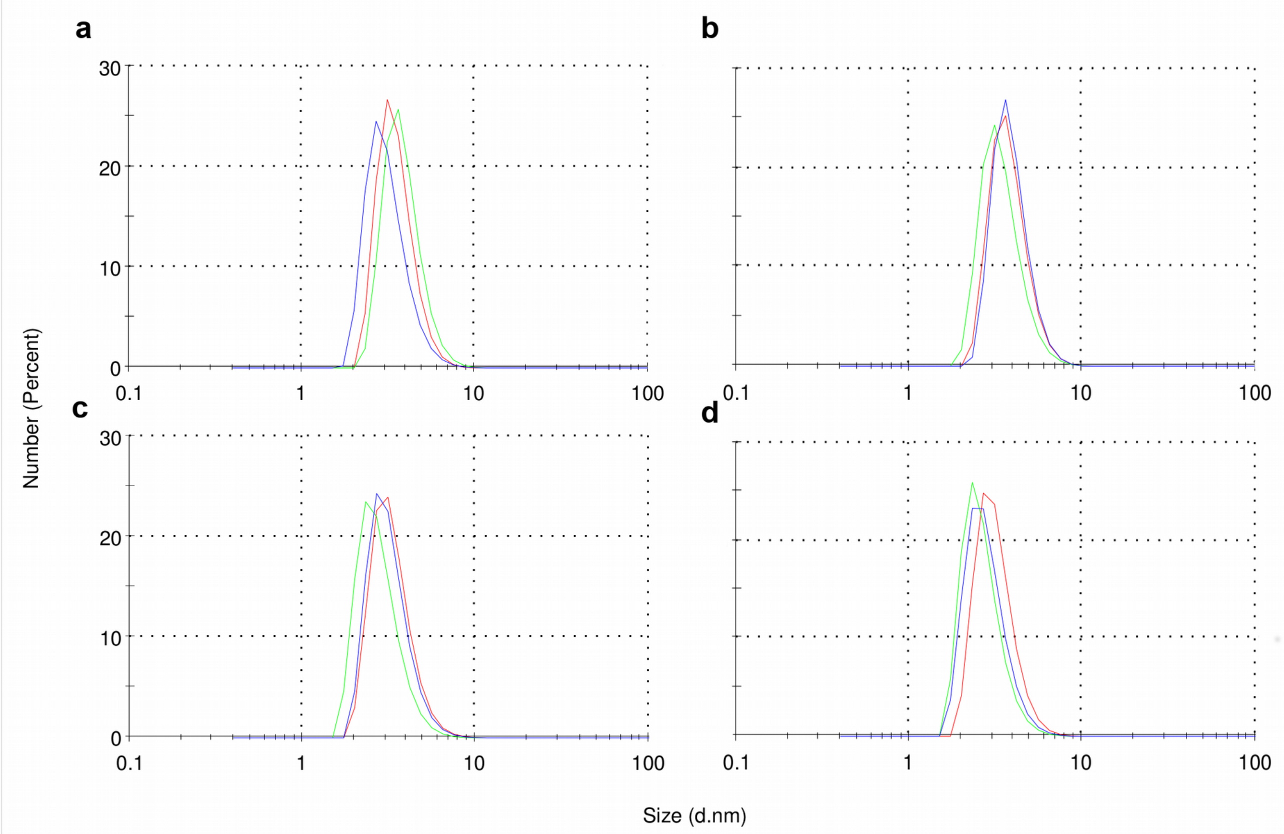

Supplementary Figure 3. Dynamic Light Scattering profiles of native and chemically oxidized Hha. Scattering profiles were measured in matched samples of Hha in which **a,c)** one was used as a control while **b,d)** the second one was treated with 5 mM H_2O_2. Measurements were taken in triplicate after **(a,b)** 2 hours and **(c,d)** 18 hours of incubation. Under these conditions, NMR spectra showed a drastic loss of the native tertiary structure but no significant differences are seen by DLS. The average estimated hydrodynamic radius (assuming a spherical particle) is 3.7 ± 0.9 nm for the control and 3.2 ± 0.7 nm for the oxidized sample, all of them in agreement with the dimensions of monomeric Hha. Thus, no significant aggregation seems to take place in the presence of H_2O_2.

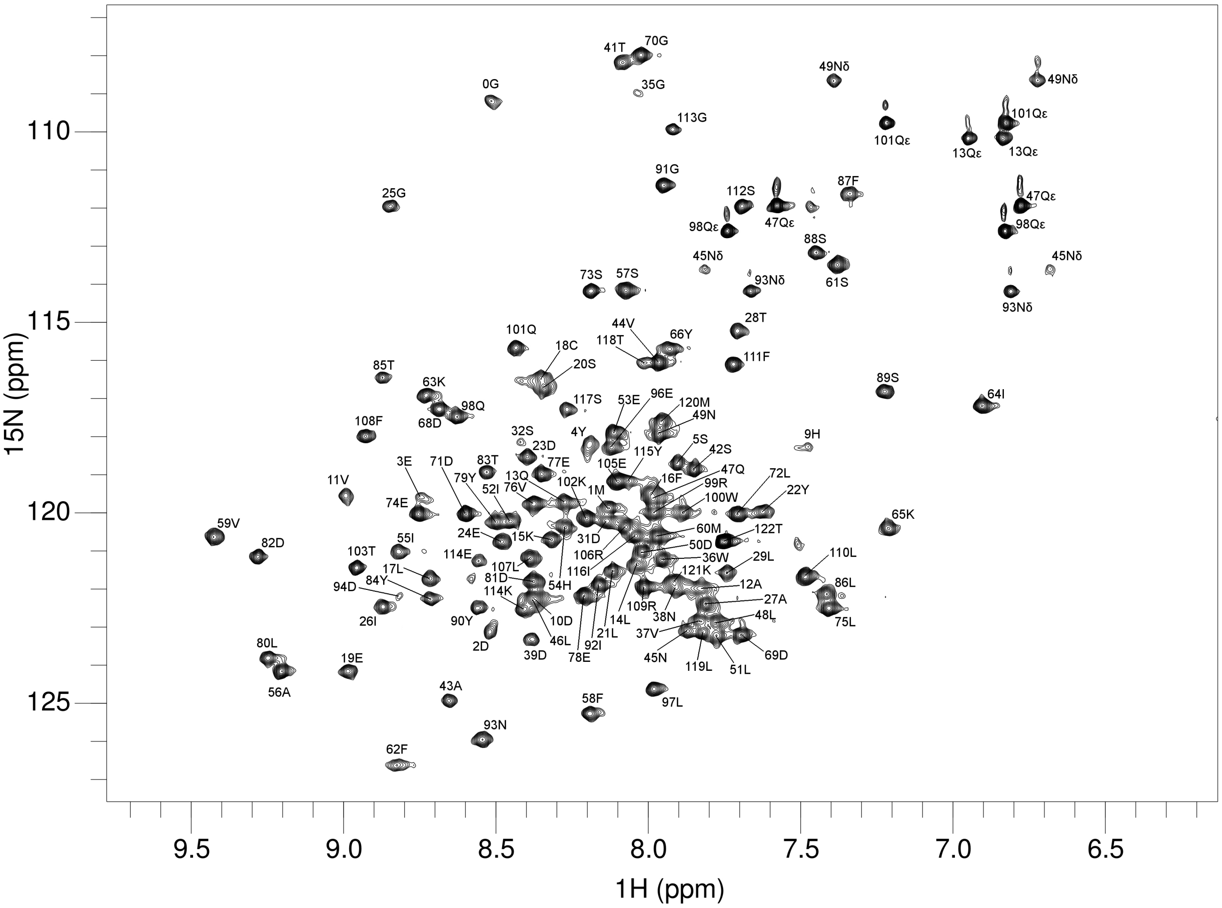

Supplementary Figure 4. ^{1}H-^{15}N HSQC-NMR spectrum of 100 µM [C117S]YmoB in 20 mM [NaH_2PO_4 + Na_2HPO_4], 150 mM NaCl, 0.2mM EDTA, pH = 7.0 with assignments indicated. Full assignments are deposited in RCSB with accession number 104115, PDBID 2MN2.

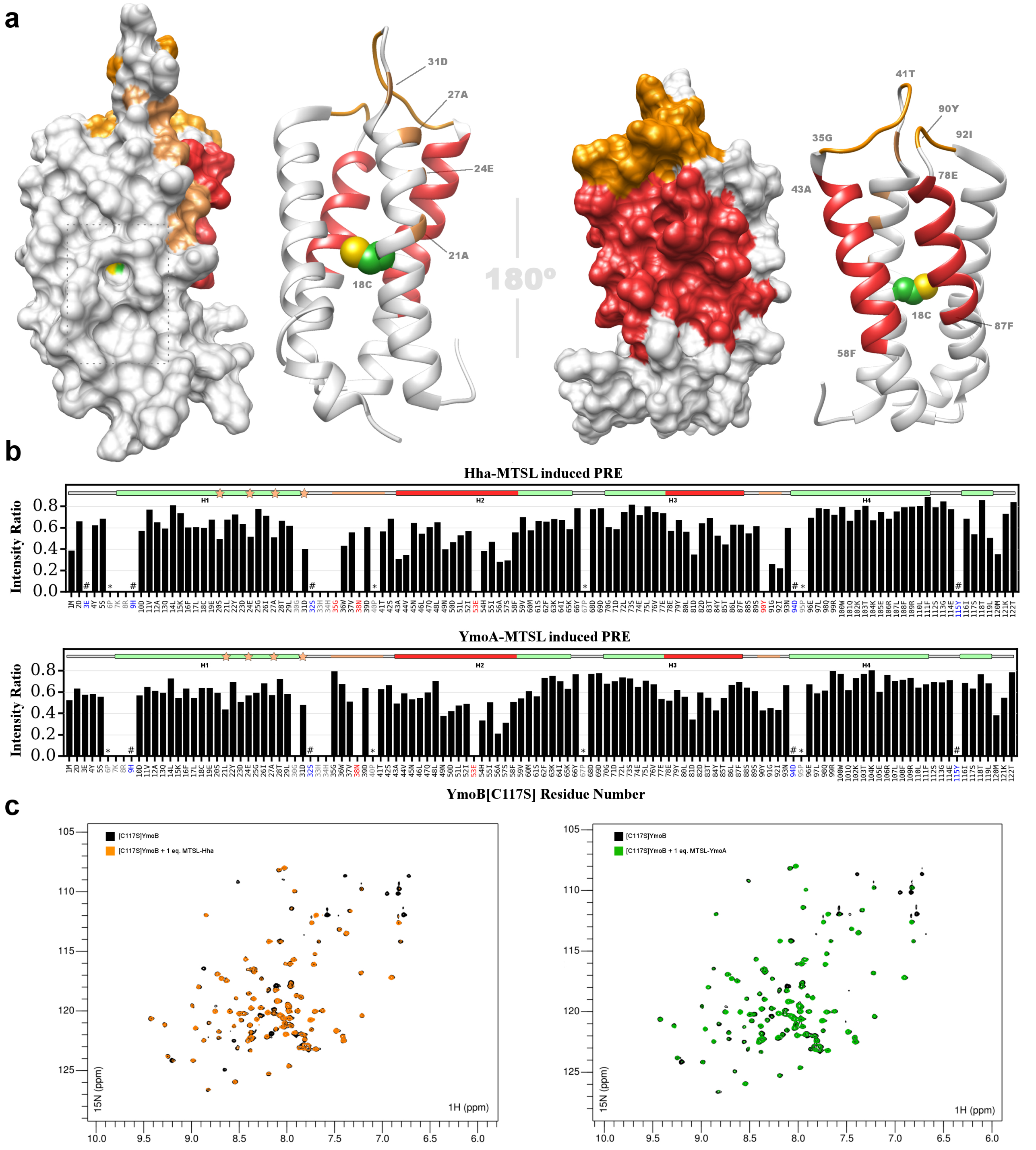

Supplementary Figure 5. Paramagnetic Relaxation Enhancement (PRE) NMR experiments. **a)** Residues with broadened NMR backbone signals in the presence of MTSL-Hha are mapped on the surface and ribbon representations of [C117S]YmoB. The affected residues in helices 2 and 3 are marked in red. Other affected residues are colored orange. The position of C18 is indicated in colored spheres. **b)** The intensity ratios of individual residues of ^{15}N-labeled [C117S]YmoB in the presence and in the absence of 1 equivalent of MTSL-Hha (top) or MTSL-YmoA (bottom) are represented. The helical regions of YmoB are indicated. The regions of helices 2 and 3 that are

more affected by the paramagnetic proteins are marked in red in the secondary structure scheme. Additional perturbed regions are marked in orange. Residues whose signal disappears upon addition of MTSL-derived partners are marked in red. Non-assigned residues are marked in grey and prolines are marked with asterisks. Residues with weak or overlapping signals are colored in blue and indicated by '#'. **c)** Supperposition of ^{1}H-^{15}N HSQC-NMR spectra of 100μM [C117S]YmoB (in black) and [C117S]YmoB in the presence of one molar equivalent of MTSL-Hha (left-orange) and MTSL-YmoA (right-green).

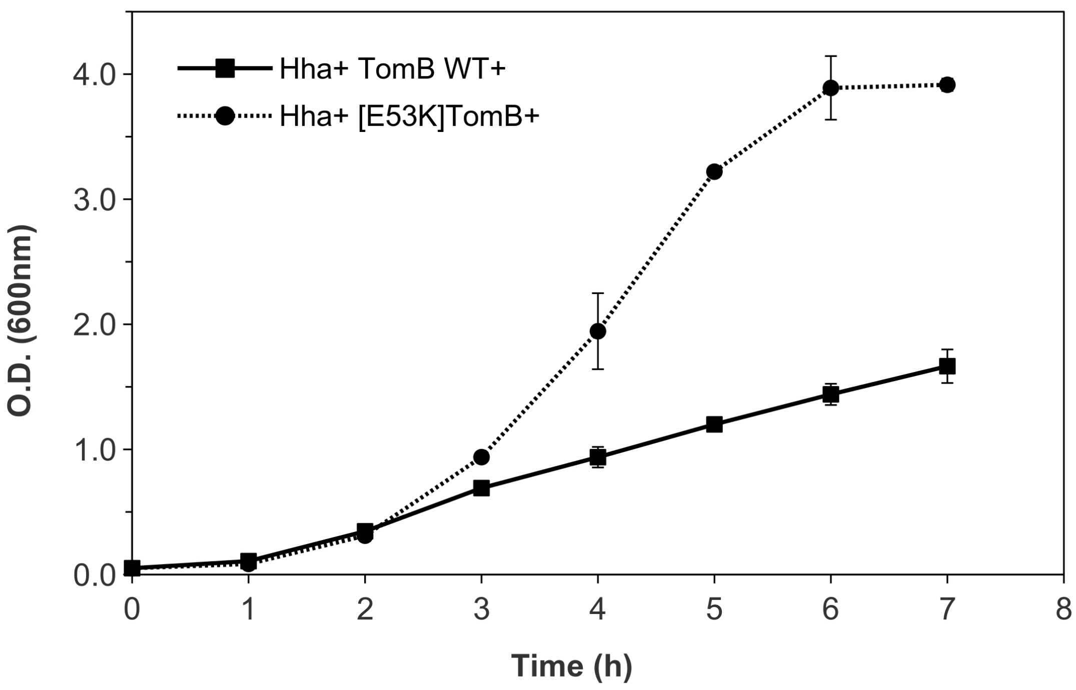

Supplementary Figure 6: Residue E53 in the Hha transient binding site is important for TomB antitoxin activity. Growth curves of *E. coli ΔhhaΔtomB* cells transformed with pCA24N-*tomB*-*hha* plasmid, WT TomB or [E53K]TomB plus Hha. Residue 53 is a conserved residue (see Figure 5d) and sits in the center of the identified Hha transient binding site in [C117S]YmoB.

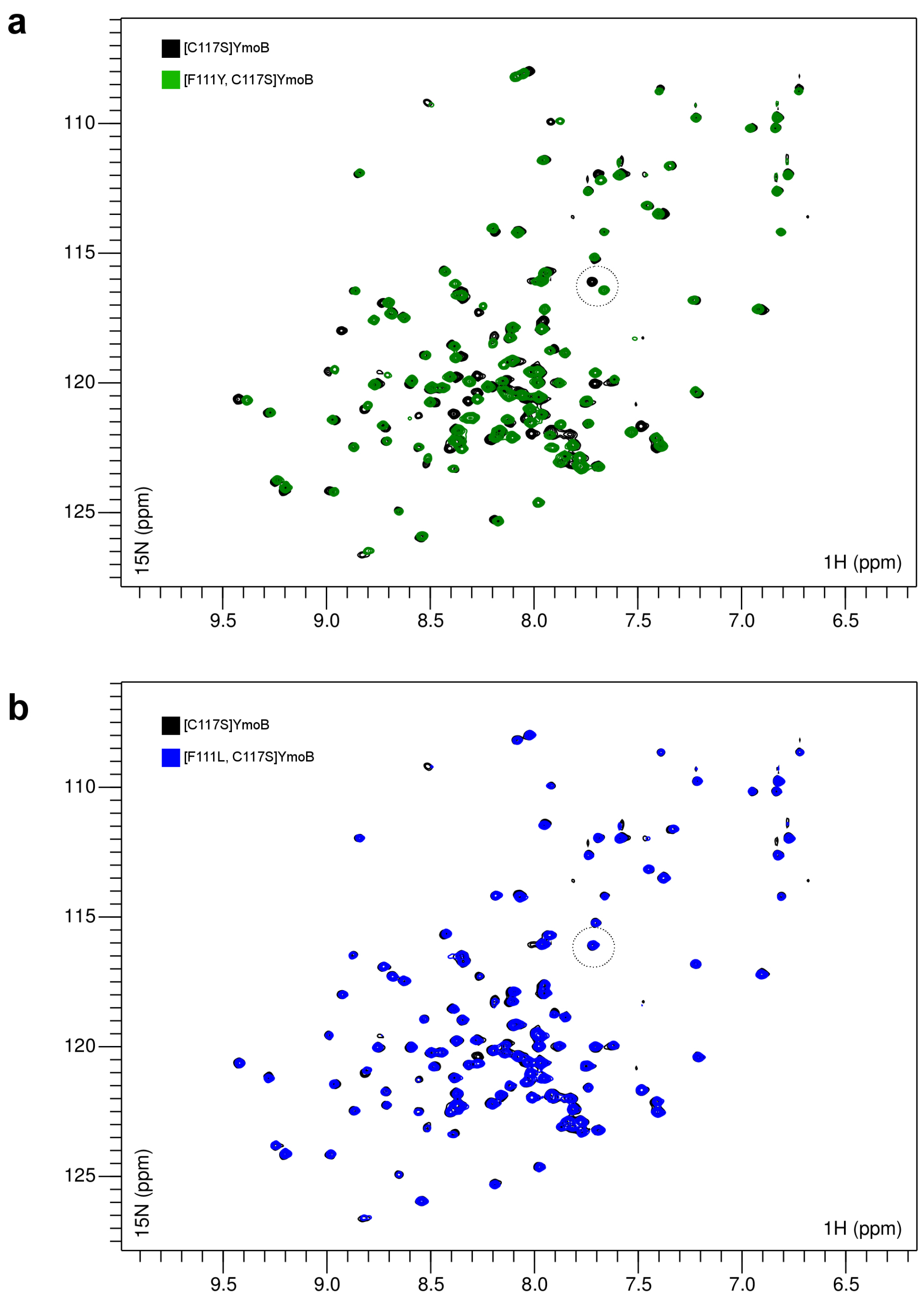

Supplementary Figure 7: ^{1}H-^{15}N HSQC-NMR spectra of **a)** [F111Y, C117S]YmoB (green) and **b)** [F111L, C117S]YmoB (blue) are typical of well folded proteins – overlaid with the spectrum of [C117S]YmoB (black). Position of residue 111 in all the three spectra is highlighted with dotted circle in both experiments. The chemical shifts of the variants with tyrosine and leucine at position 111 are very similar suggesting little deviations from the WT structure.

Detected peptide	Detected mass (amu)	-SH	-SOH	$-SO_2H$	$-SO_3H$	Dimedone adduct
**YmoB/ [C117S]YmoB**						
[11]VAQLKFLcE[19]	1050.562	○				
	1066.559		○			
	1082.556			○		
	1098.552				○	
[115]YIcTLmKT[122]	972.489	○				
	988.484	●	○			
	1004.479		●	○		
	1020.474			●	○	
	1036.470				●	
	1110.561					○
	1126.555					●
**YmoA**						
[17]KcTTIDTLER[26]	1179.603	■				
	1195.598		■			
	1211.592			■		
	1227.588				■	
	1317.673					■
**Hha**						
[18]cQTIDTLER[26]	1078.518	■				
	1094.519		■			
	1110.513			■		
	1126.504				■	

Supplementary Figure 8. Cysteine oxidized peptides detected by MS. YmoB/[C117S]YmoB peptides generated by GluC endopeptidase. [115]YIcTLmKT[122] peptides appear only in WT YmoB. The relevant cysteine oxidation state is indicated. In methionine containing peptides the cysteine oxidation state may be ambiguous. Empty and filled circles correspond to the assignments if methionine is unmodified or oxidized, respectively. YmoA/Hha unambiguous C18 oxidations were obtained by trypsin digestion. Deviations between experimental and theoretical masses were lower than 3.94 ppm.

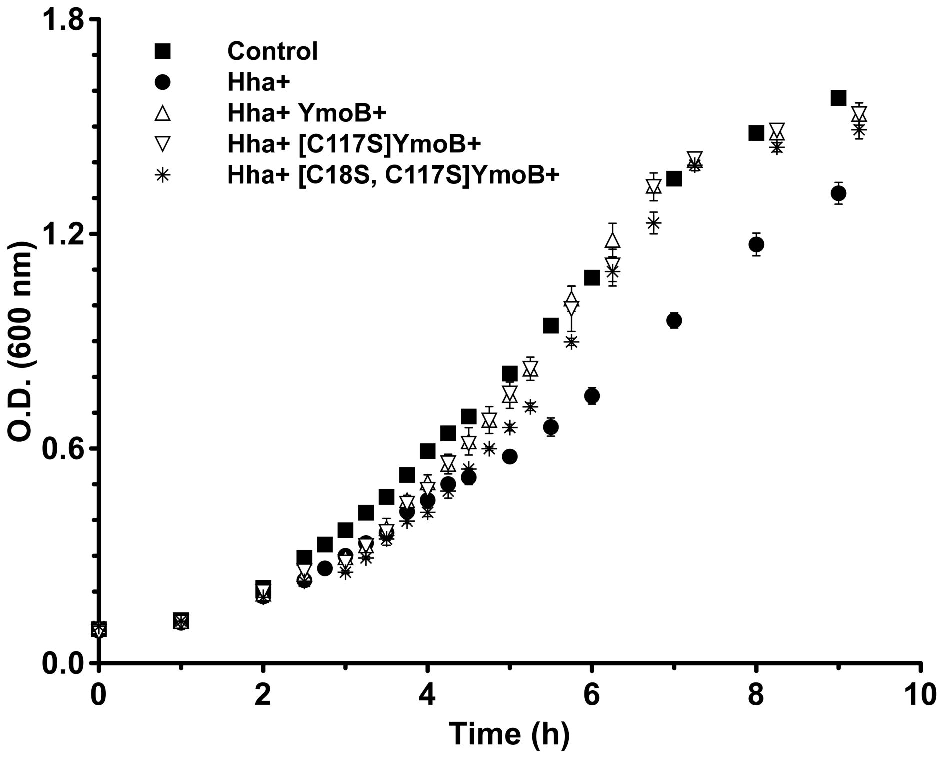

Supplementary Figure 9. Cysteine free variants of YmoB retain antitoxin activity. Growth curves of *E. coli* K12MG1655 Δhha cells harboring pCA24N-hha and pBAD30-ymob, pBAD30-ymob(C117S) or pBAD30-ymob(C18S)(C117S). Control cultures with no overexpression of Hha or any antitoxin (squares) were compared with those overexpressing: Hha (circles), Hha and YmoB, (triangles up), Hha and [C117S]YmoB (triangles down) or Hha and [C18S, C117S]YmoB (asterisks). Experiments were performed at 37ºC in Triptone Minimal Medium in duplicate. The mean and sample standard deviation are shown.

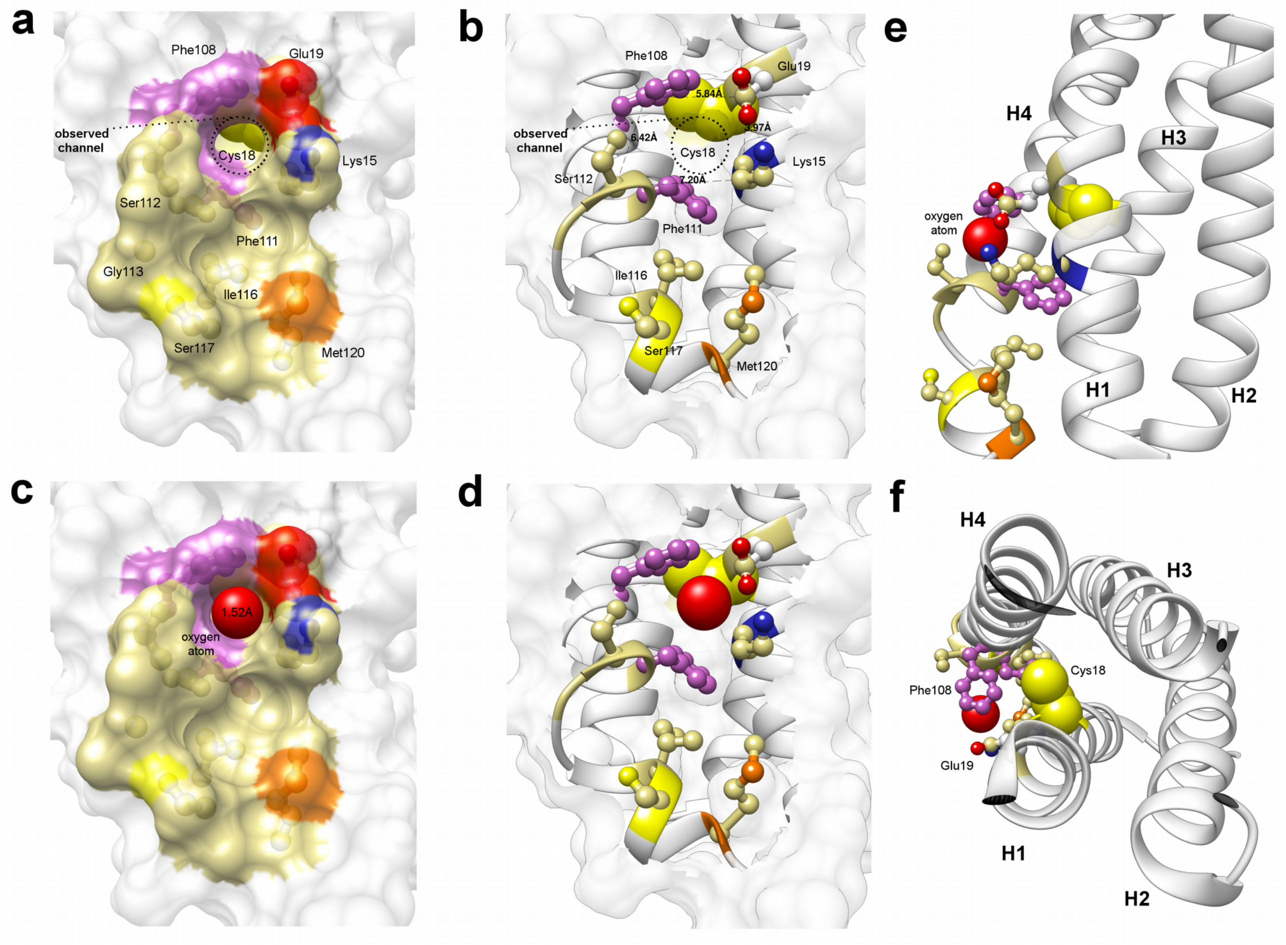

Supplementary Figure 10: Zooming into the channel of YmoB. **a)** surface representation of the channel as in Figure 5c. **b)** The same representation showing the side chains of relevant residues in ball and stick representation, and C18 as solid spheres. **c)** and **d)** The same representations as and b but including a red sphere with the approximate size of an oxygen molecule. **e)** and **f)** Orthogonal views of panel **d)**.

Supplementary Table 1: CFU analysis of TomB antitoxin activity dependency on oxygen (See Figure 1).

	**OD600**	**CFU**	**CFU/OD**	**Approx. %O2 Sat.**
**Control**	0.05	325	6144	90%
	0.23	169	750	-
	0.84	>300	*	1%
	1.37	>300	*	10%
**Hha+**	0.06	221	3576	90%
	0.15	127	820	-
	0.52	237	455	40%
	1.01	124	**123**	10%
**Hha+TomB+**	0.07	121	1798	90%
	0.14	84	613	-
	0.54	162	301	1%
	1.16	>300	*	10%

CFU were obtained by plating 10 µL of a 10^8 dilution of samples taken at the indicated optical densities. The last column gives the ratio between CFU and OD. Asterisks indicate that the number of CFU was higher than 300.

CFU/OD ratios of the starting cultures, with very low OD, are much smaller for the protein overexpressing cells. This may reflect the additional metabolic burden. However, in the stationary phase with 10% oxygen saturation, the number of CFU of the cells overexpressing Hha and TomB is larger than those only expressing Hha, ruling out that the observed toxic effect of Hha is just related to the metabolic stress related to protein overexpression.

Supplementary Table 2: NMR experiments used to assign backbone atoms H-N-C', side chain atoms C^{α}/C^{β} and H^{α}/H^{β}.

Experiment	Dimensionality	Observed Spins
^{1}H-^{15}N-HSQC (H_2O and D_2O)	2D: ^{1}H-^{15}N	H_i-N_i
HNCO	3D: ^{1}H-^{15}N-^{13}C	H_i-N_i-$C'O_{-i}$
HNCACO	3D: ^{1}H-^{15}N-^{13}C	H_i-N_i-$C'O_{-i}$-$C'O_i$
CBCAcoNH	3D: ^{1}H-^{15}N-^{13}C	H_i-N_i-C^{α}_{-i}-C^{β}_{-i}
CBCANH	3D: ^{1}H-^{15}N-^{13}C	H_i-N_i-C^{α}_{i}-C^{β}_{i}-C^{α}_{-i}-$C\beta_{-i}$
HBHAcbcacoNH	3D: ^{1}H-^{15}N-^{1}H	H_i-N_i-H^{α}_{i}-H^{β}_{i}-H^{α}_{-i}-H^{β}_{-i}
^{1}H-^{13}C-HSQC-CT 10% [^{13}C]-glucose* (in D_2O)	2D: ^{1}H-^{13}C	H_i-C_i aliphatic

Supplementary Table 3: Interleaved-NUS NMR experiments recorded to assign aromatic and aliphatic side chain atoms and to obtain distance restrains based on NOESY experiments.

	SPARSE %	nt	d1	Acquisition time (ms)			Total time, h
				^{1}H-^{1}Hind	^{13}C	^{15}N	
900 MHz spectrometer							
NOESY-CHSQC ali	33	4	1	70/36	24	-	68.5
NOESY-NHSQC	30	4	1	70/20	-	64	42
NOESY-CHSQC aro	33	8	1.3	70/36	35	7	53.4
800 MHz spectrometer							
CCH-TOCSY	50	4	1.5	79/17	8.5	-	53
HCCH-TOCSY	50	4	1.5	79/17	20	-	60
